# Integrated transcriptomics and metabolomics analysis to characterize cold stress responses in *Nicotiana tabacum*

**DOI:** 10.1186/s12864-017-3871-7

**Published:** 2017-06-29

**Authors:** Jingjing Jin, Hui Zhang, Jianfeng Zhang, Pingping Liu, Xia Chen, Zefeng Li, Yalong Xu, Peng Lu, Peijian Cao

**Affiliations:** China Tobacco Gene Research Center, Zhengzhou Tobacco Research Institute of CNTC, Zhengzhou, 450001 China

**Keywords:** Transcriptome, Metabolome, Tobacco, Cold stress

## Abstract

**Background:**

CB-1 and K326 are closely related tobacco cultivars; however, their cold tolerance capacities are different. K326 is much more cold tolerant than CB-1.

**Results:**

We studied the transcriptomes and metabolomes of CB-1 and K326 leaf samples treated with cold stress. Totally, we have identified 14,590 differentially expressed genes (DEGs) in CB-1 and 14,605 DEGs in K326; there was also 200 differentially expressed metabolites in CB-1 and 194 in K326. Moreover, there were many overlapping genes (around 50%) that were cold-responsive in both plant cultivars, although there were also many differences in the cold responsive genes between the two cultivars. Importantly, for most of the overlapping cold responsive genes, the extent of the changes in expression were typically much more pronounced in K326 than in CB-1, which may help explain the superior cold tolerance of K326. Similar results were found in the metabolome analysis, particularly with the analysis of primary metabolites, including amino acids, organic acids, and sugars. The large number of specific responsive genes and metabolites highlight the complex regulatory mechanisms associated with cold stress in tobacco. In addition, our work implies that the energy metabolism and hormones may function distinctly between CB-1 and K326.

**Conclusions:**

Differences in gene expression and metabolite levels following cold stress treatment seem likely to have contributed to the observed difference in the cold tolerance phenotype of these two tobacco cultivars.

**Electronic supplementary material:**

The online version of this article (doi:10.1186/s12864-017-3871-7) contains supplementary material, which is available to authorized users.

## Background

Low temperature is one of the key environmental stresses that many plants have to cope with during their life cycle [1–3]. It can influence growth and development, as well as the yield, quality, and geographic distribution of crop plants. Cold stress can be classified as either chilling stress (0–15 °C) or freezing stress (<0 °C) [1, 4]. Generally, plants from temperate regions have the ability to cold acclimate, that is, to develop increased freezing tolerance after being exposed to low, nonfreezing temperatures [4]. However, many important crops, such as rice, maize, soybean, cotton, tomato, and tobacco, which originated in the tropics or subtopics, lack a cold acclimation mechanism and are thus sensitive to cold stress [5]. Conventional cross-breeding has used the cold-tolerant varieties as the main source to increase the cold tolerance of less tolerant cultivars [6]. However, the current lack of information about the detailed molecular mechanisms of cold stress biology limits the potential improvement of major crops [5].

Following the rapid progress in DNA microarray and sequencing technologies, whole transcriptome analysis is now being used frequently for transcriptome profiling [7–11]. Many studies investigating plant responses to cold stress have been reported [12–17]. Numerous cold-responsive genes have been identified from different species, including *Arabidopsis thaliana* [2, 18], *Oryza sativa* [19–22], *Zea mays* [23, 24], *Prunus persica* [25, 26], and many other species [13, 27–30]. At present, the best-understood genetics network associated with a role in cold acclimation is the Arabidopsis CBF cold response pathway [31]. Exposing Arabidopsis plants to low temperature results in rapid induction of a small family of genes that encode transcriptional activators known as C-repeat/dehydration responsive element (*DREB*)-binding factors (e.g. CBF1, −2, −3, −4, −6 or DREB1b, −c) [31]. These genes encode transcription factors that belong to the AP2/ERF domain family of DNA-binding proteins [32]. Furthermore, cold transcriptome analysis has shown that other cold regulatory gene networks also contribute to freezing stress tolerance and cold acclimation [33, 34]. Cold stress signaling integrates with various other signaling pathways to coordinate stress responses with plant growth and development. Cold can affect the expression of genes involved in gibberellin (*GA*) signaling [35]. Regulation of auxin homeostasis is known to be involved in cold stress responses. Several key components of the cytokinin signaling are also known to play roles in cold signaling. In addition to phytohormones, both the circadian clock and the physiological changes associated with the developmental transition to flowering are known to interact with cold signaling [2]. Despite these insights, many cold acclimation traits still cannot be explained based only on the differential expression change of single or multiple genes. Hence, much remains to be elucidated in the complicated gene network and in its interactions with various biomolecules including metabolites.

In addition to their complex gene regulatory networks, plants have multiple highly-regulated metabolic networks that can play important roles in their growth under cold treatment [34]. It is well established that cold treatment elicits the expression and/or activity of many biochemical pathways. Fox example, sugars and amino acids are widely accepted as related to cold response, as they are not only vital for the synthesis of functional proteins but also serve as precursors for a large array of metabolites with multiple functions in plant responses to cold treatment [31]. There have been only a limited number of metabolite studies conducted that seek to uncover the potential links between gene and metabolite networks during cold treatment.

Recent technical advances in genome sequencing, gene expression profiling, and metabolomics have made it possible to dissect the complex processes involved in plant responses to cold treatment [7, 8, 19, 33, 36, 37]. Using an integrated gene/metabolite approach, Hirai et al. and Monika et al. were able to identify new genes involved in glucosinolate metabolism in response to sulfur depletion [7, 38], and Tohge et al. (2005) identified novel flavonoid biosynthesis pathways in transgenic Arabidopsis plants overexpressing a MYB transcription factor [8]. Moreover, Kaplan et al. (2007) revealed sequential changes in both gene expression and metabolite profiles throughout the course of the cold acclimation process [37]. Thus, research using multiple-omics tools can potentially lead to the identification of useful genes that could help to improve yields in plants that must cope with cold environments.

Tobacco (*Nicotiana tabacum*) is native to tropical and subtropical America, but it is now commercially cultivated worldwide. Moreover, it is sensitive to cold [18, 29]. Hence, the molecular mechanism of cold response has been of great concern in the production of this crop. Jin et al. tried to use proteomics to identify the important proteins which may be related to cold response in tobacco [39]. However, owing to the proteomic technique, only 101 proteins were found in their studies. Several works tried to increase the cold tolerance of tobacco using genes from other plants [18, 40]. However, the candidates were still very limited.

Here, our objectives were to explore, on a large scale, the changes that occur in the transcriptome and the metabolome of tobacco in response to low temperature. Moreover, we sought to reveal potential links between cold response genes and metabolites. The experiments that comprised this study included a comparison of the transcriptome and metabolite profiles of two tobacco ecotypes, CB-1 and K326; CB-1 was found to be more sensitive to low temperature than K326. Taken together, our results indicate that cold acclimation is associated with extensive changes in both the transcriptome and metabolome. In general, both the common cold-response genes and the common cold-responsive metabolites between CB-1 and K326 had a greater degree of change in K326 than in CB-1. The specific differentially accumulated transcripts/metabolites and different trends of secondary metabolites in K326 and CB-1 suggest that several cold responsive pathways exist in tobacco. In addition, energy metabolism may be different, and hormones appear to have distinct roles in CB-1 vs. K326.

## Methods

### Plant materials and cold treatments

The *Nicotiana tabacum* ecotypes K326 and CB-1 were used in this study. For the RNA-seq experiments, seeds were first germinated and cultivated in plastic pots under typical conditions until the plants grew to the six-leaf stage. Control plants were then grown in a growth chamber under normal conditions (28 °C with 16 h light and 24 °C with 8 h dark). For cold treatments, plants were transferred to 4 °C for one day with afore mentioned photoperiod; the one day treatment duration was chosen because the cold marker gene *ICE1* was found in a pilot study to reach a maximum of expression after one day of such treatment (Additional file 1: Figure S1). Samples were harvested one day after the start of cold treatment (light period). The top two leaves of each plant were sampled. All leaf samples were immediately frozen in liquid nitrogen and stored at −80 °C. Each sample consisted of leaves from six plants grown in the same condition.

### RNA isolation and library preparation for transcriptomics analysis

Total RNA was isolated using TRIzol reagent (Invitrogen) according to the manufacturer’s instructions. Poly (A) + mRNA was purified with oligo (dT) beads, and then the mRNA was randomly segmented into small fragments by divalent cations in fragmentation buffer (Illumina) at 94 °C for 5 min. These short fragments were used as templates to synthesize first-strand cDNA using random hexamer primers. Second-strand cDNA was synthesized using RNaseH and DNA polymerase I. Short cDNA fragments were purified with a QiaQuick PCR extraction kit (Qiagen). The cDNA fragments were then connected with sequencing adapters according to an Illumina protocol. After agarose gel electrophoresis, the target fragments of 300–500 bp were selected for PCR amplification to create the final cDNA library.

### Sequencing, assembly, and functional annotation

The RNA libraries were prepared using the TruSeq RNA Sample Preparation Kit v2, set A (RS-122-2001, Illumina) according to the product instructions [9–11]. All experiments were repeated two times. The quality and size of the cDNA libraries for sequencing were checked using the Agilent 2200 TapeStation system (Agilent). The libraries were run on single lanes for 100 cycles (paired-end) on HiseqTM 2000 (illumine Inc.), individually. Raw reads were analyzed by FastQC (http://www.bioinformatics.babraham.ac.uk/projects/fastqc/) for quality, and high-quality reads with Q > 20 were obtained using *NGSToolkits* (version: 2.3.3) [41]. The Trinity method was used for de novo assembly of the high-quality reads to generate unigenes [42]. Functions of the unigenes were annotated based on sequence similarities to sequences in the public UniProt database [43]. T-test was used for the statistics analysis.

### DNA preparation and qRT-PCR

For cDNA preparation, approximately 1 μg of total RNA from each sample was used to synthesize first-strand cDNA in a 20 μl reaction solution using a Transcriptor First-Strand cDNA Synthesis Kit (Roche). The transcript levels for transcripts related to cold response were detected using quantitative real-time PCR (qRT-PCR) [9, 10]. The qRT-PCR amplification reactions were performed using a LightCycler ® 96 Real-Time PCR System (Roche). The reactions were conducted in a final volume of 20 μl containing 10 μl of SYBR Green premix (2X) (Roche). The PCR thermocycling program consisted of an initial incubation at 95 °C for 10 min, followed by 40 cycles of 95 °C for 10s, 58 °C for 20s, and 72 °C for 20s, and an additional final cycle for analysis of dissociation curves to ensure specific amplification. Relative quantification of the transcription level for each target gene was normalized to the signal for transcripts of the constitutively expressed tobacco *26S* gene. T-test was used for statistic significant analysis. The gene specific primers used for qRT-PCR are listed in Additional file 1: Table S1.

### GC-MS sample preparation

The freeze-dried tissue were ground to a uniform powder, filter using a 40-mesh sieve, and stored at -80 °C until the metabolic study. The tissue powder(20 mg)was added to a 2 mL Eppendorf tube and soaked in 1.5 mL of an extraction solvent containing isopropanol/acetonitrile/water(3/3/2,*v*/v/v) with 15 μL (2 mg/mL) of tridecanoic acid as an internal standard. All extracts were sonicated for 1 h and centrifuged for 10 min (1,4000 rpm, 4 °C). Then the supernatant (1.3 mL) was removed to a new 1.5 mL centrifuge tube. After centrifugation at 1,5000 rpm for 10 min, 4 °C, 350 μL of supernatant was collected to a conical insert of a 2 mL glass vial and dried under nitrogen flow on an N-EVAP Nitrogen Evaporator. The silylationreation for increasing the volatility of the metabolites was performed by adding 100 μL of methyl-trimethyl-silyl-trifluoroacetamide(MSTFA) to the sample and incubating it for 60 min at 60 °C.

### GC/MS data analysis

Metabolomic analysis was performed on an Agilent 7683B series injector (Agilent, Santa Clara, CA) coupled to an Agilent 6890 N series gas chromatograph system and a 5975 mass selective detector (MSD) (Agilent, Santa Clara, CA). Aglient DB-5MS column (0.25 μm, 0.25 mm × 30 m, Agilent Technologies, Inc., Santa Clara, CA) was used. The column temperature was 70 °C for the first 4 min and then increased at 5 °C/min to 310 °C for 15 min.The injection temperature was set as 300 °C, and the injection volume was 1 μL with a 10:1 split ratio. Helium (99.9995%, China) was applied as a carrier gas. The column flow was 1.2 mL/min, and the column was equipped with a linear velocity control model. Prior to the instrumental analysis, the mass spectrometer was tuned using perfluorotributylamine (PFTBA) to obtain optimum performance. A simultaneous full scan-selected ion monitoring mode (Scan-SIM) was used to acquire the data. The mass spectra scanning scope was set to 33–600 m/z in the full scan mode and 90 chromatographic peaks of 25 groups were set in the selected ion monitoring. The scan speed is 2.59 scan s-1 and the solvent cut time is 5.0 min. The temperatures of the interface and the ion source were adjusted to 280 and 230 °C, respectively. The detector voltage was maintained at 1.2 kV, and the electron impact (EI) model was selected to achieve ionization of the metabolites at 70 eV.

### LC-MS sample preparation

The freeze-dried leaf tissues were ground to a uniform powder, filtered with a 40-mesh sieve, and stored at -80 °C. 2 mg tissue powder was added to a 2 mL Eppendorf tube to which was added 1.5 mL of an extraction solvent containing a 75% methanol with 10 μg/ml Umbelliferon as an internal standard. All extracts were sonicated for an hour and were centrifuged for 10 min (14,000 rpm, 4 °C). 1 ml of supernatant was collected for LC − MS analysis.

### LC-MS data analysis

LC − MS analysis was performed on an Agilent 1290 lipid chromatograph system and a 6540 6540 UHD Q-TOF equipped with an electrospray ionization source (Agilent, Santa Clara, CA). Aglient SB-C18 column (RRHD,1.8 μm, 2.1 × 100 mm, Agilent Technologies, Inc., Santa Clara, CA) was used. The samples were analyzed in positive ion mode. The gradient was as follows: 2 min, 30% B; 10 min, 85% B; 15 min, 90% B; 17 min, 100% B; 20 min, 100% B. Mobile phases A and B were 95% water: 5% acetonitrile with 0.1% formic acid and 5% water: 95% acetonitrile with 0.1% formic acid, respectively. The flow rate was 0.3 mL/min. 5 μL aliquots of sample were loaded for every individual analysis. The capillary voltage and spray shield were set to 3500 V. The sheath gas was set to 10 L/min at a temperature of 350 °C. The neb gas was set to 12 L/min at 350 °C. Spectra were acquired over the m/z 50–1200 range. The collision energy in the MS/MS mode was set to 20 V.

### Gene network construction and visualization

The interaction between genes was retrieved from several public protein-protein interaction (PPI) databases, including BioGrid [44], IntAct [45], BOND [46], DIP [47], MINT [48], HPRD [49], MIPS [50], and TAIR [51]. The interaction between genes and metabolites was calculated as Pearson-correlation coefficients, with a threshold value of 0.9 for inclusion in the network. Cytoscape [52] was used to visualize the final interaction network.

## Results

### Phenotypic and physiological responses to cold stress

**Fig. 1:**
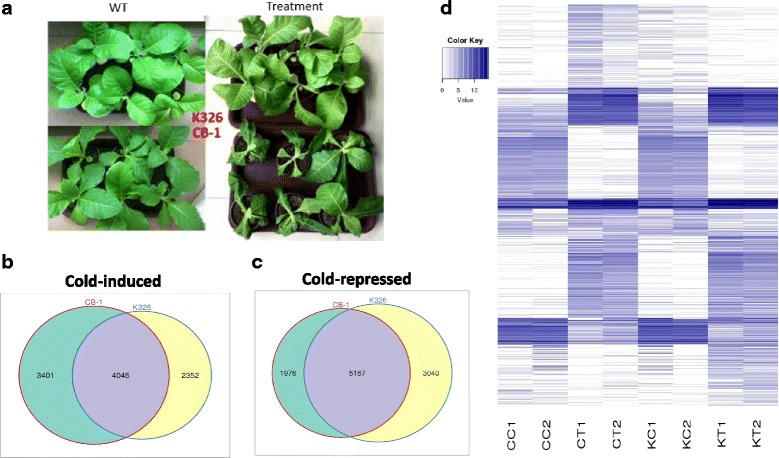
Overview of transcriptome analysis. **a** Phenotype for K326 and CB-1 under cold treatment. Left Panel: untreated plants; Right Panel: Plants after cold treatment. Up Panel: Wildtype and cold treated K326 plants; Down Panel: Wildtype and cold treated CB-1 plants. **b** Venn graph for CB-1 and K326 based on cold up-regulated (induced) genes. **c** Venn graph for CB-1 and K326 based on cold down-regulated (repressed) genes. **d** Heatmap of DEG expression in CC, CT, KC, and KT

The leaf tissues of CB-1 and K326 were chosen to study cold responses. The phenotype of CB-1 and K326 was checked after one day of cold treatment. From the phenotype shown in Fig. 1a, it is clear that K326 is much more cold-tolerant than CB-1, for which more plants survived the cold treatment. Totally, 10 out of 12 CB-1 samples were nearly dead, whereas around 2–3 out of 12 K326 samples were nearly dead (Fig. 1).

### Transcriptome analysis for CB-1 and K326

We used RNA-seq to enable comparison of the transcriptomes of CB-1 and K326 in response to cold treatment. Around 100 million high quality reads of 90 base pairs (bp) were generated for each library. Table 1 shows an overview of the RNA-seq reads for the eight libraries; a total of 664,432,026 high-quality reads were obtained from all libraries. Using the Trinity method with default parameters [42], these high-quality reads were finally assembled into 135,148 unigenes with 230,735 isoforms. All unigenes were longer than 200 bp. The N50 of the final assembled transcripts was 1479 bp. For an overview of the assembly results refer to Additional file 2: Data Set 1.1. The unigenes were annotated by performing BLASTX searching against the UniProt database [43]. Among the 135,148 non-redundant unigenes, 23,246 had at least one hit via BLASTX [53] searching with an E-value <= 1e-3 and at least 30% sequence identity, highlighting that there are still many uncharacterized genes or tobacco specific genes. In order to calculate the expression level for the assembled transcripts, we first mapped reads onto assembled unigenes using bowtie [54]. RSEM (RNA-seq by Expectation-Maximization) [55] was used to estimate the abundance of assembled transcripts and to calculate the expression level.

**Table 1:** Summary of sequencing data quality

Sample	reads	HQ reads	Total bps	Total bps in HQ reads	Q20 (%)	N (%)	GC (%)
CT1	55,643,444	52,340,970	5,619,987,844	5,286,437,970	98.52	0.89	42.54
CT2	117,283,874	110,687,372	11,845,671,274	11,179,424,572	98.48	1.02	42.86
CC1	90,303,872	85,210,840	9,120,691,072	8,606,294,840	98.53	0.89	43.81
CC2	102,439,604	96,792,568	10,346,400,004	9,776,049,368	98.53	0.89	43.11
KT1	81,708,490	77,877,970	8,252,557,490	7,865,674,970	98.87	0.89	42.46
KT2	85,728,846	81,281,390	8,658,613,446	8,209,420,390	98.78	0.85	42.59
KC1	61,961,226	58,117,664	6,258,083,826	5,869,884,064	98.73	0.85	42.53
KC2	107,703,362	102,123,252	10,878,039,562	10,314,448,452	98.72	0.87	43.10
total	702,772,718	664,432,026	70,980,044,518	67,107,634,626	98.65	0.89	42.88

CC refers to the control group of the CB-1 sample; CT refers to the cold treatment group of the CB-1 samples; KC refers to the control group of the K326 samples; KT refers to the cold treatment group of the K326 samples; 1 and 2 refers to the two replicates

**Fig. 2:**
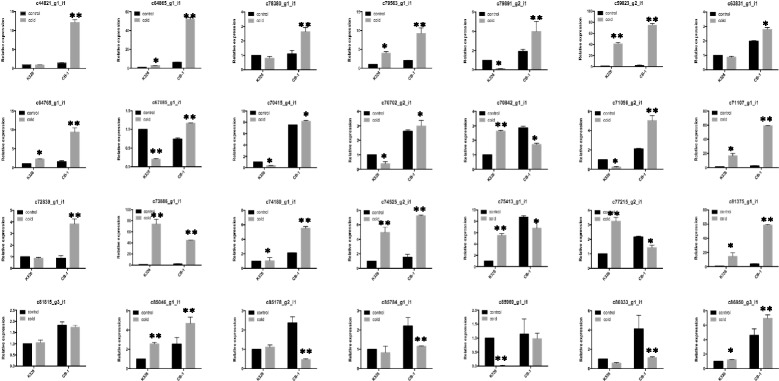
qRT-PCR analyses of unigenes. c44821_g1: MYB; c64865_g1: CRF6; c78380_g1: WRKY33; c79563_g1: DREB1D; c79891_g2: ERF109; c59823_g2: unknown; c63831_g1: ELF4; c64765_g1: DREB1F; c67085_g1: ARR6; c70415_g4: NFYB7; c70702_g2: DREB1B; c70842_g1: COR413; c71056_g1: DREB2H; c71107_g1: BHLH92; c72839_g1: ARR4; c73886_g1: DREB2A; c74180_g1: GAI; c74525_g2: ABR1; c75413_g1: RAV1; c77215_g2: unknown; c81375_g1: ERF053; c81815_g3: ARR14; c85046_g1: CMTA3; c85178_g2: AHK3; c85784_g1: HOS1; c85969_g1: AHK4; c86033_g1: ARR1; c86950_g3: SIZ1. ** means *p*-value < 0.01 between treatment and control; * means *p*-value < 0.05 between treatment and control

To verify the RNA-seq data, 28 genes were chosen for qPCR validation, including *DREBs*, *CORs*, and *SIZ1*, which have already been reported to be related to cold responses. The results of the RNA-seq and qPCR analyses were similar (Fig. 2), showing the same general expression trends (more than 90% correlation), such as DREB (c79563_g1).

### Identification of differentially expressed genes under cold stress

Differentially expressed genes (DEGs) among samples were defined using fold change values by the expression of assembled transcripts. We defined DEGs with |log2(fold change) > 1| and corrected *p*-value < 0.05. In total, we identified 7447 up-regulated DEGs between the control and cold-treated CB-1 samples, with 7143 down-regulated DEGs. For K326, there were 6398 up-regulated DEGs, with 8207 down-regulated DEGs (Fig. 1b, c). Gene expression profiles in CB-1 and K326 changed under cold stress to differing degrees; however, there were some overlapping DEGs that were regulated by cold stress in both tobacco cultivars, with 41.3% for up-regulated DEGs and 50.7% for down-regulated DEGs. To compare the transcriptomes in CB-1 and K326 under cold treatment, a heat map was generated to present the transcript abundance for all DEGs under cold stress (Fig. 1d). The results also showed that a series of changes in gene expression in CB-1 and K326 when plants subjected to cold treatment.

### Cold response genes common between CB-1 and K326

Following cold treatments, 4046 cold-induced transcripts and 5167 cold-repressed transcripts were identified in both CB-1 and K326 (Fig. 1b, c). We analyzed these groups, trying to investigate the mechanisms that function in responses to cold stress. Functional classification of DEGs was achieved using a gene ontology (GO) analysis via InterproScan [56]. These DEGs were assigned to three classes of GO: biological processes, molecular functions, and cellular compartments. Additional file 1: Figure S2 illustrates the distinct distribution of the main GO categories. The GO terms “response to stimulus”, “signaling” and “metabolic process” are the most highly enriched among both the up-regulated and down-regulated gene sets, which is in agreement with previous findings [17, 57, 58]. GO terms also represented diverse functional activities corresponding with the mentioned biological processes. The highest numbers of DEGs were categorized in “catalytic and binding activities”, which have also been reported as over-represented terms under treatment with a variety of stresses [16, 33]. In contrast with previous studies, which highlighted the role of ABA-related events under cold stress [1], no such events were detected in our study. Interesting, the GO terms “immune system process”, “negative regulation of biological process”, and “growth” were enriched in the GO list of cold down-regulated genes.

**Fig. 3:**
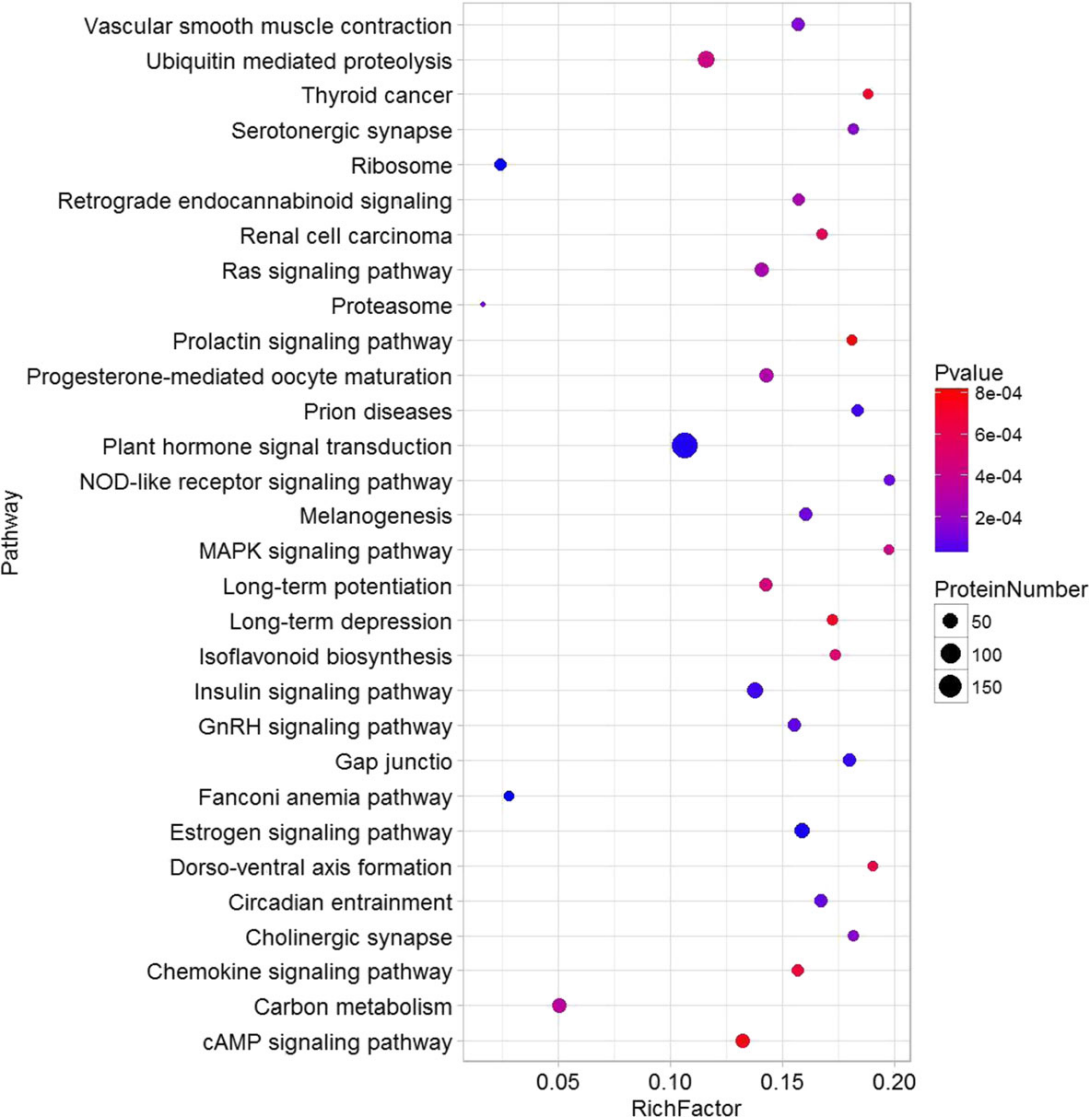
Top 30 enriched pathways for overlapping cold responsive genes between K326 and CB-1

KEGG (Kyoto Encyclopedia of Genes and Genomes) pathway analysis provides classifications that are valuable for studying the complex biological functions of genes [17]. KEGG analysis showed that “Ribosome”, “Plant hormone signal transduction”, and “Estrogen signaling pathway” were significantly enriched by DEGs under cold treatment (Fig. 3, Additional file 2: Dataset 1.2). “MAPK signaling pathway” and “cAMP signaling pathway” were also enriched. Previously, it was reported that different protein kinase families (such as MAPKs) are activated by osmotic stresses [1, 2, 33]. These protein kinases may be important components in the signal transduction pathway of various environment signals in plants under cold stress.

Furthermore, by checking the detailed expression levels for these DEGs, many genes were induced to much higher in K326 than CB-1, especially for known cold related genes such as the DREB genes (Additional file 1: Table S2, Fig. 1d). This may be one of the major reasons that K326 is much more cold-tolerant than CB-1 as indicated by our gross phenotypic analysis.

### Cultivar specific differentially expressed genes (DEGs)

**Fig. 4:**
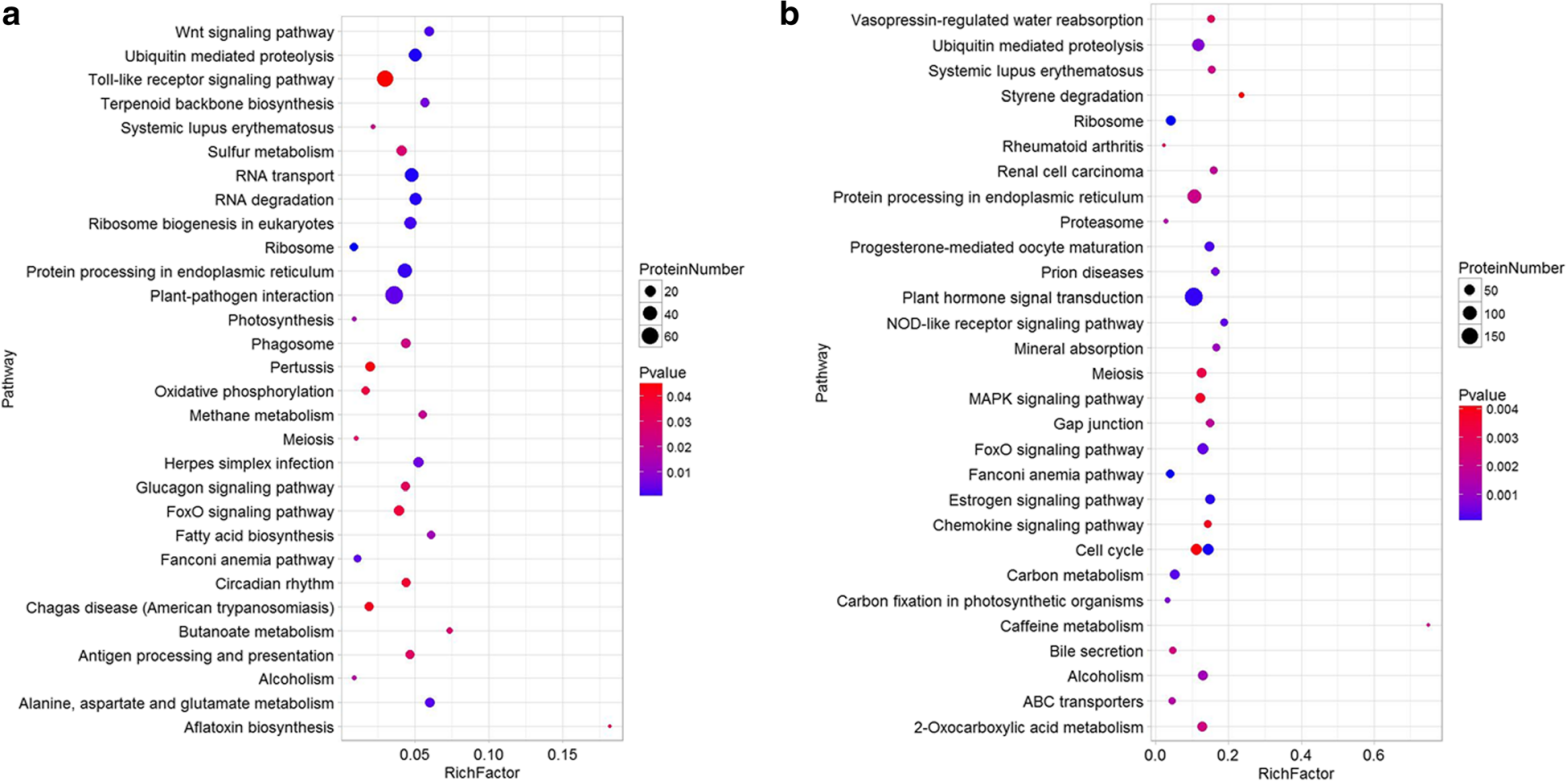
Top 30 enriched pathways for specific cold responsive genes of CB-1 and K326. **a** Top 30 enriched pathways for CB-1 specific cold responsive genes. **b** Top 30 enriched pathways for K326 specific cold responsive genes

In total, 5377 DEGs were only identified in CB-1 in the cold treatment, with 3401 up-regulated and 1976 down-regulated (Fig. 1b, c). And 5392 DEGs were identified in K326, with 2352 up-regulated and 3040 down-regulated (Fig. 1b, c). Similar with common DEGs between CB-1 and K326, KEGG enrichment analysis for the K326-specific DEGs revealed that “Ribosome”, “Plant hormone signal transduction”, and “MAPK/Estrogen/FoxO signaling pathway” were highly enriched, which suggested the ability for cold tolerance of K326 was strengthened (Fig. 4b). In addition, K326-specific DEGs were enriched in “2-Oxocarboxylic metabolism”, “Caffeine metabolism”, “Carbon metabolism” in K326, which CB-1-specific DEGs were enriched in “Alanine, aspartate and glutamate metabolism”, “Butanoate metabolism”, and “Methane metabolism” in CB-1. (Fig. 4a, b, Additional file 1: Figure S3).

### Metabolome reprogramming under cold treatment in CB-1 and K326

**Fig. 5:**
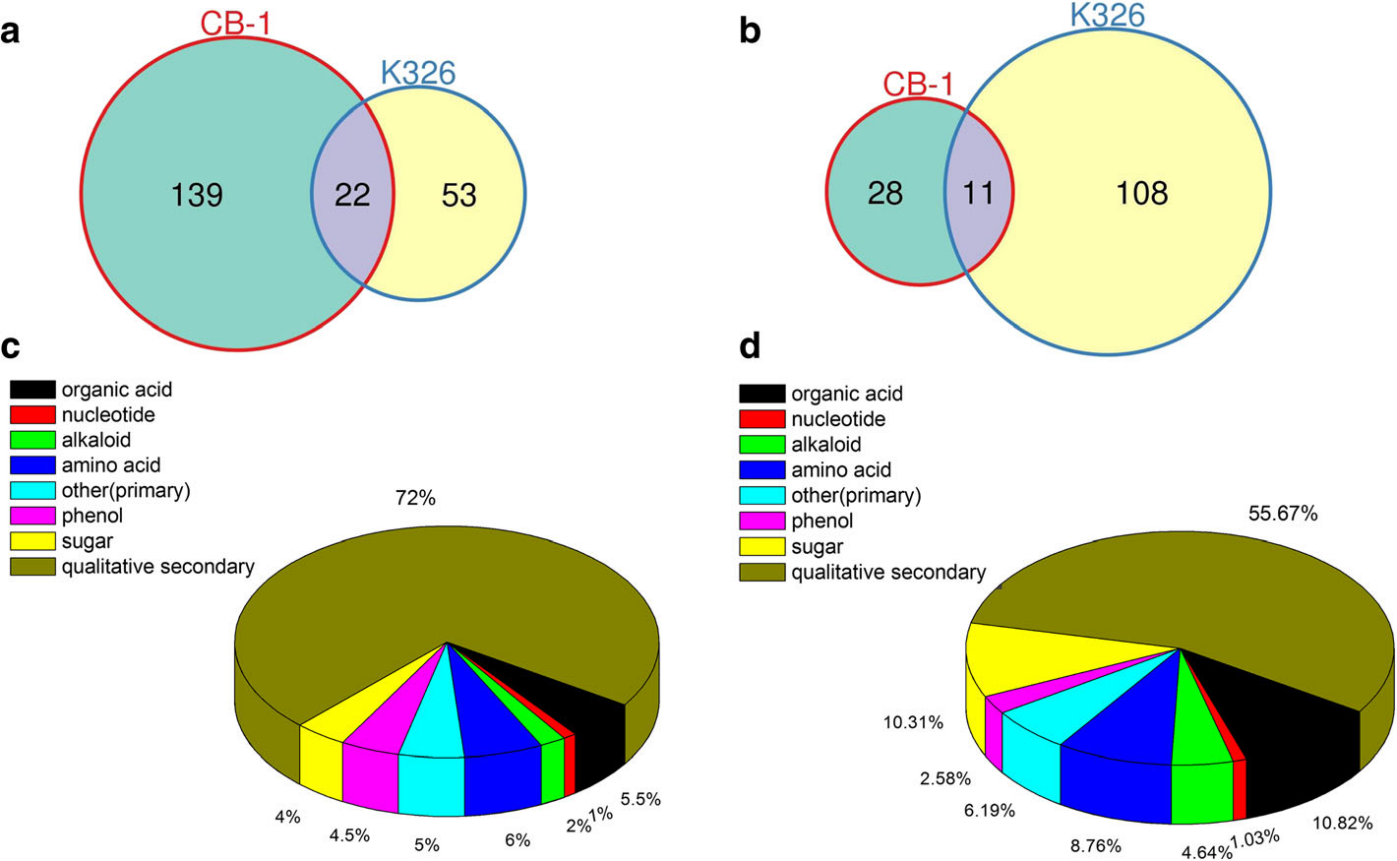
Classification of identified metabolites. **a** Venn graph for up-regulated differentially accumulated metabolites between CB-1 and K326. **b** Venn graph for down-regulated differentially accumulated metabolites between CB-1 and K326. **c** Pie graph for differentially accumulated metabolites in CB-1. **d** Pie graph for differentially accumulated metabolites in K326

In an attempt to generate a comprehensive picture of metabolite reprogramming that occurs in response to cold treatment, we performed metabolite profiling of CB-1 and K326 using GC-MS and LC-MS. We reproducibly detected a total of 198 primary metabolites and 4269 secondary metabolites, with 4045 un-target metabolites and 224 target metabolites (Additional file 2: Dataset S1.3). Among these identified primary metabolites and secondary metabolites, 200 metabolites showed differential accumulation (Fold change >1.5) in CB-1, with 161 increased and 39 decreased under cold treatment compared with the control (Fig. 5a, b). In contrast, 194 differentially accumulated metabolites were identified in K326, with 75 increased and 119 decreased (Fig. 5a, b).

Of the metabolites with differential accumulation during cold treatment, several of them had previously been shown to increase in Arabidopsis plants upon exposure to low temperature [31], including several amino acids, which was found in our data (Fig. 5c, d). However, these amino acids increased to a much greater extent in K326 than in CB-1; several of them, including glycine, phenylalanine, and alanine, even decreased in CB-1 (Additional file 2: Dataset S1.3). Similar results were also found for sugars (glucose, fructose, inositol, galactinol, raffinose, and sucrose); and trehalose, putrescine, and ascorbate (Additional file 2: Dataset S1.3) [31, 37]. As in the transcriptome data, these changes may help to explain how it is that K326 is much more cold-tolerant than CB-1.

**Fig. 6:**
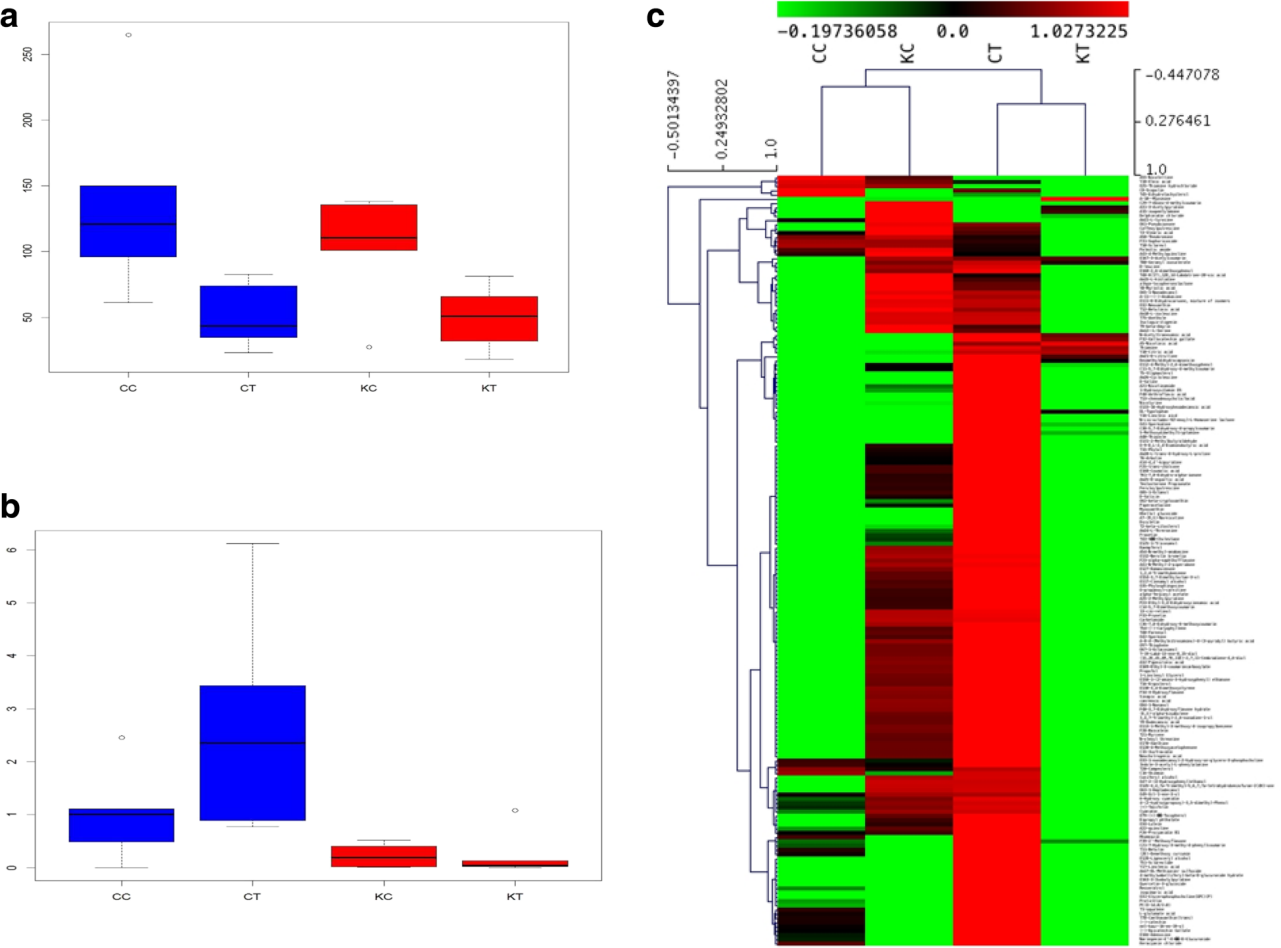
Different patterns for CB-1 and K326 based on the identified secondary metabolites. **a** Boxplot for ethylene levels in CC, CT, KC, and KT. **b** Boxplot for salicylic acid levels in CC, CT, KC, and KT. **c** Heatmap of differentially accumulated secondary metabolites for CC, CT, KC, and KT

Plant hormone signals appear to have different influences on CB-1 and K326. Unfortunately, ABA was not detected in our study. However, ethylene and salicylic acid (SA) were among the differentially-accumulated metabolites. Ethylene is negatively correlated with the cold tolerance of both CB-1 and K326 (Fig. 6a), which has already been reported in previous studies [35]. In contrast, different from previous reports, SA was in our results positively correlated with CB-1, whereas it was negatively correlated with K326 (Fig. 6b).

Another interesting finding is that we observed a lot of differential accumulation of secondary metabolites. Besides previously-reported metabolites such as flavonoids and lipid related metabolites, a fragrance (benzene) and nicotine (nicotine, Nicotinic acid) were also found to be regulated by cold treatment in our study. However, most of the identified secondary metabolites were up-regulated by cold stress in CB-1, while K326 showed the opposite trend (Fig. 6c, Additional file 1: Figure S4). More work will need to be done to investigate the reasons for these differences.

**Fig. 7:**
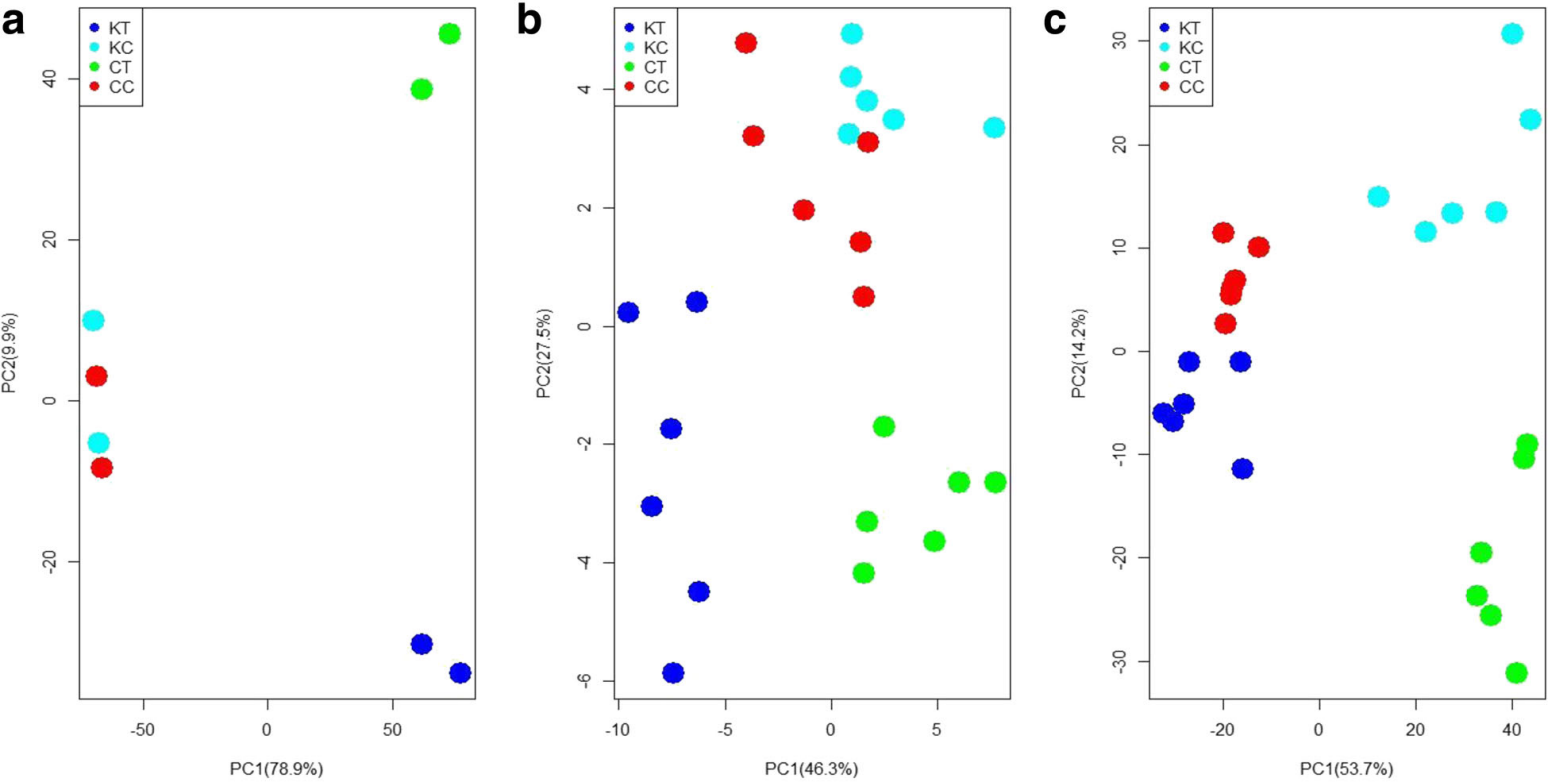
A parallel principal component analysis (PCA) of gene and metabolite profiles under cold treatment. Each point represents one experiments for gene expression and metabolite profiling. **a** PCA model for gene expression experiments. **b** PCA model for primary metabolite profiling. **c** PCA model for secondary metabolite profiling

In a PCA model based on differentially accumulated genes and metabolites, the first two principal components clearly separated all samples, both control and cold treatment for both CB-1 and K326, and explained around 80% of the total variation in the entire data set (Fig. 7). Specifically, PC1 of transcriptome and metabolism analysis may reflect cold-responsive genes and metabolites, most of which are shared by CB-1 and K326, such as amino acids and sugars. However, PC2 may largely reflect the reprogrammed parts between CB-1 and K326, especially on secondary metabolites (Fig. 7).

### Interaction network analysis between cold regulated genes and metabolites

Gene-metabolite interaction networks can be used to help understand functional relationship and to aid in identifying new regulatory elements [33]. Here, we constructed an interaction network based on the differentially expressed genes (Fig. 1b, c) and metabolites (Fig. 5a, b). Gene-gene interactions were retrieved from known PPI databases. All genes harboring potential interaction relationships are shown in Additional file 2: Dataset S1.4 (Additional file 1: Figure S5).

From the clusters shown in Additional file 1: Figure S5, it is apparent that most of the secondary metabolites closely connect together, including flavonoid families. In order to check the interaction relationships, we also showed the detailed networks for sugar metabolites (Additional file 1: Figure S6A) and target secondary metabolites (Additional file 1: Figure S6B). Furthermore, the sugar-related network can be divided into two clusters, one with sucrose, trehalose, tagatose, and another with cellobiose, fructose, and raffinose. The interaction relationships in these two sugar clusters suggest not only that the *CBF/DREB* and *ZAT* families play some role with the biosynthesis of sugar metabolites, but also suggest that TFs, including those of the ARR, GT, UPBEAT, VIP, and MYB families, may also contribute to sugar biosynthesis during plant responses to cold treatment. These potential new markers for cold stress could be useful for cold resistance studies in the future.

### Analysis of cold-responsive transcription factors

Transcription factors (TF) play a key role in the regulation of gene expression under abiotic and biotic stresses in plants. RNA-seq results showed that many TFs were regulated under cold stress in CB-1 and K326 (Additional file 2: Dataset S1.5, Table 2). In total, more than 20 families of TFs were differentially expressed, implying their important roles in regulating genes involved in cold responses.

**Table 2:** Top 30 differentially expressed transcription factors (TF) under cold treatment

gene_id	CC	CT	KC	KT	FC(CB-1)	FC(K326)	name
c78380_g1	0.733	6.628	1.314	7.238	5.895	5.925	WRKY33
c64765_g1	0.000	2.709	0.000	5.693	2.709	5.693	DREB1F
c74525_g2	0.000	5.085	0.344	5.873	5.085	5.530	ABR1
c40491_g1	0.000	2.973	0.035	5.456	2.973	5.421	HBP-1b
c77368_g4	0.000	4.180	0.000	5.395	4.180	5.395	HSFA4B
c78380_g2	1.097	5.904	1.211	6.379	4.807	5.168	WRKY33
c64865_g1	0.000	4.614	0.000	5.127	4.614	5.127	CRF6
c79891_g2	0.743	2.194	0.496	5.479	1.452	4.982	ERF109
c81375_g1	0.567	4.541	0.358	5.193	3.975	4.834	ERF053
c74235_g2	0.195	3.704	0.345	4.962	3.509	4.617	WRKY40
c83950_g2	0.180	3.968	0.397	4.863	3.788	4.466	WRKY6
c73886_g1	0.000	2.621	0.000	4.253	2.621	4.253	DREB2A
c72670_g1	1.984	5.685	2.190	6.424	3.701	4.234	WRKY11
c71056_g2	0.000	2.704	0.000	4.217	2.704	4.217	DREB2H
c52393_g1	0.588	4.436	0.000	4.165	3.847	4.165	DREB2H
c69854_g3	2.768	6.470	2.503	6.573	3.702	4.070	NAC002
c44821_g1	0.288	3.422	0.000	3.913	3.135	3.913	MYB306
c81178_g1	0.000	2.706	0.000	3.904	2.706	3.904	ABR1
c83320_g1	0.470	3.990	0.473	4.347	3.519	3.874	WRKY42
c112787_g1	4.060	0.506	4.140	0.301	−3.554	−3.839	UPBEAT1
c58192_g1	0.000	3.259	0.000	3.814	3.259	3.814	HSFA6b
c73618_g1	0.000	2.962	0.000	3.714	2.962	3.714	GT-3A
c72172_g1	1.011	4.721	1.536	4.261	3.710	2.725	HSFC1
c69854_g2	1.541	5.161	1.664	5.352	3.621	3.688	NAC002
c81178_g3	0.000	2.275	0.000	3.649	2.275	3.649	RAP2–6
c68026_g1	3.934	0.320	4.060	0.792	−3.613	−3.268	TCP19
c69854_g1	1.792	4.978	2.347	5.835	3.187	3.487	NAC002
c67824_g2	1.961	4.702	1.968	5.439	2.741	3.470	WRKY40
c76796_g2	0.000	1.908	0.139	3.517	1.908	3.378	WRKY41
c82962_g2	4.150	1.453	4.123	0.783	−2.697	−3.341	MYB39

The value is log2 RESM value for each assembled TF. FC is the log2 fold change in the treatment sample as compared to wild type

In particular, families showing especially strong responses to cold treatment included WRKY, AP2-EREBP, NAC, MYB, HSF, bHLH, UPB, and bZIP (Additional file 2: Dataset S1.5, Table 2). For instance, some genes from these families were up-regulated by more than 30 times during cold treatment. For example, most of our predicted CBF TFs presented dramatic activation (especially c64765_g1 with nearly 60 times up-regulation); this gene is known to play a direct role in activating the expression of downstream cold-responsive genes, except for c71296_g1. Another is a famous known cold regulator *ICE1*, which encodes a MYC-type basic helix-loop-helix (bHLC) TF [2, 3] that directly binds to MYC cis-elements in the promoter of CBF/DREBs. The RNA-seq results showed that the transcript level of only one MYC-type bHLH (c71107_g1) increased under cold stresses, suggesting that *DREBs* may act as its target.

## Discussion

**Fig. 8:**
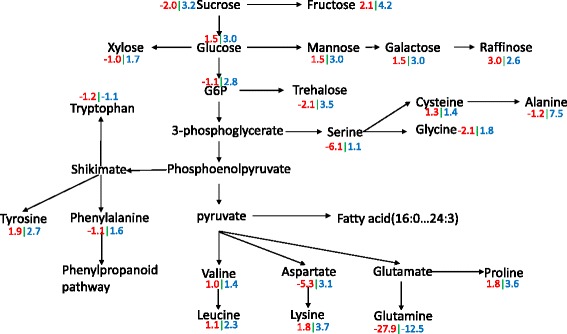
The energy metabolism through sugar and amino acids metabolic pathways under cold treatment. *Red color* represents the fold change between wild type and cold stress in CB-1. *Blue color* represents the fold change between wild type and cold stress in K326

**Fig. 9:**
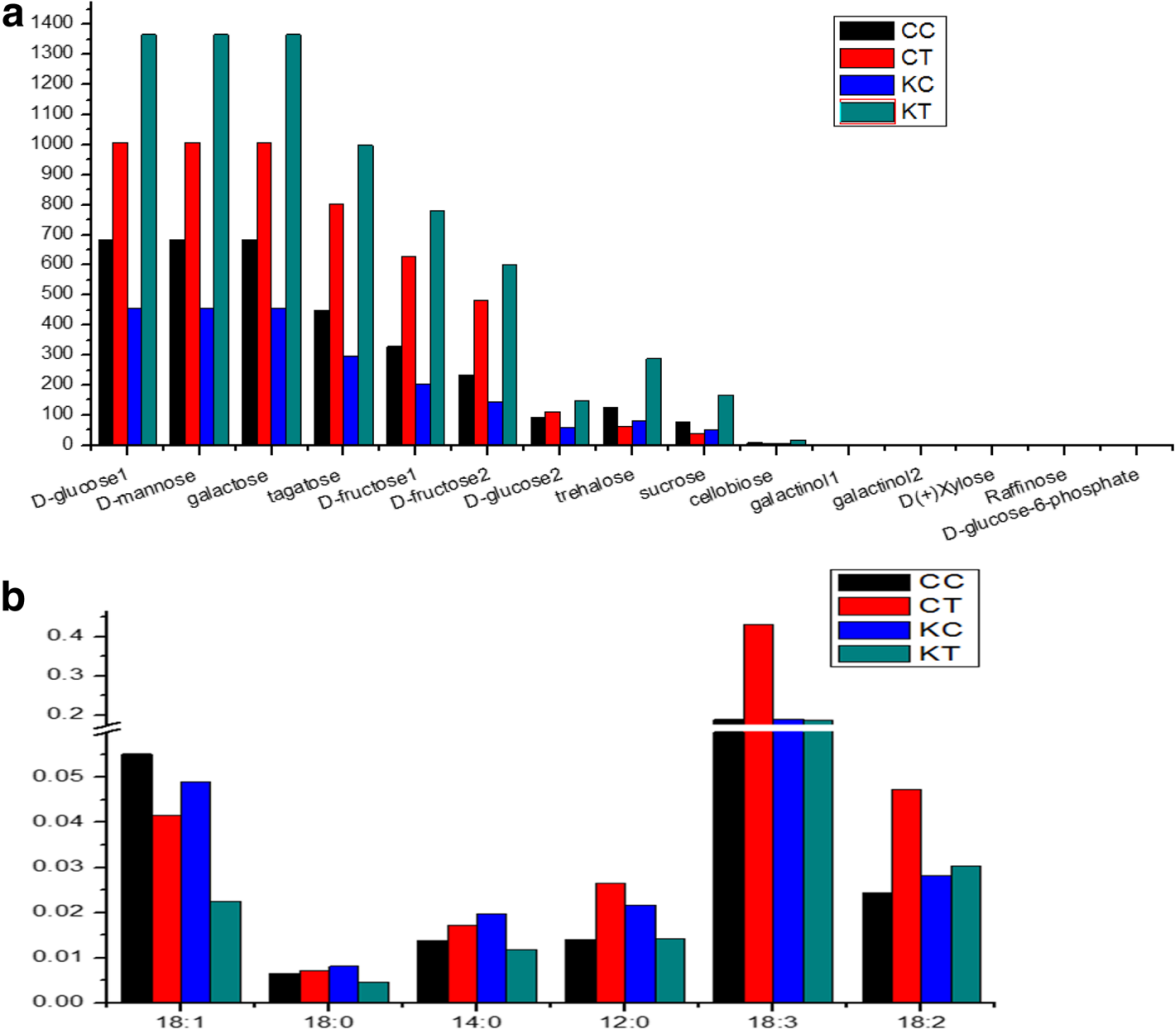
The levels of sugar metabolites and fatty acid metabolites. Y-axis represents the relative quantitate level of metabolites. **a** The relative metabolite levels for sugars. **b** The relative metabolite levels for fatty acids

In plants subjected to cold treatment, to enhance adaptability to low temperature, more sugars and other energy carriers (e.g., ATP) are consumed to produce lipids, amino acid, membrane components, and other molecules to further promote cell membrane fluidity and structural rearrangement [19, 33]. In our study, a lot of sugar components showed up-regulation under cold treatment in both CB-1 and K326 (Figs. 8 and 9a), including glucose, fructose, inositol, galactinol, and raffinose, but excepting trehalose and sucrose in CB-1. Meanwhile, some sugars that have not been widely-reported to be related to cold responses were also detected in our study, such as tagatose, mannose, and cellobiose. It seems evident that the sugar metabolism is much more active in K326 than in CB-1 in response to cold treatment (Figs. 8 and 9a). However, for lipids, another alternative of energy metabolism, especially linolenic acid (18:3), was down-regulated in K326 but up-regulated in CB-1 (Fig. 9b). CB-1 and K326 have no significant differences in the accumulation of some amino acids including valine, leucine, and tyrosine, whereas serine and L-aspartate were accumulated to much higher extents in K326 (Fig. 8, Additional file 2: Dataset 1.6). Together, these results suggest that CB-1 and K326 may employ different aspects of energy metabolism as they respond to cold stress.

Hormones are signaling molecules that play key roles in regulating gene expression under cold stress [27, 35, 59]. RNA-seq analysis revealed that many genes related to abscisic acid, ethylene, auxin, salicylic acid, and gibberellin were regulated by cold stress in CB-1 and K326 (Additional file 2: Dataset 1.7). The KEGG pathway, “plant hormone signal transduction”, was significantly enriched among the differentially expressed genes in both CB-1 and K326.

Alternations of ethylene levels by cold stress treatment have been observed in many plants [35]. However, the role of ethylene in cold tolerance varies in different plant species, and can even vary in Arabidopsis plants that are grown in different environments [35]. For example, the cold tolerance of soil-grown Arabidopsis seedlings treated with 1-aminocyclopropane-1-caroxylated (ACC) is enhanced [60] but is reduced in vitro; cold tolerance is increased when aminoethoxyvinylgycine (AVG) is applied [61]. Ethylene positively affects the cold tolerance of tomato (*Lycopersicon esculentum*) [62]. In contrast, ethylene levels are negatively correlated with the cold tolerance of *Medicago truncatula* [27]. Previous studies have shown that ethylene also negatively influences the cold tolerance of tobacco (*Nicotiana tabacum*) [40], which was confirmed by our finding that ethylene levels are negatively correlated with the cold tolerance in both CB-1 and K326, with a greater magnitude of down-regulation in K326. Therefore, the role of ethylene on cold tolerance is possibly species dependent. ERF TFs, which are located downstream of the ethylene signal pathway, play important roles in cold responses via recognizing dehydration-responsive elements (DRE) [40, 61, 62]. This family can be further divided into ERF (Ethylene-responsive transcription factor ERF), CRF (Ethylene-responsive transcription factor CRF), ABR (Ethylene-responsive transcription factor ABR), RAP (Ethylene-responsive transcription factor RAP) and EIN (ETHYLENE INSENSITIVE protein) (Additional file 1: Table S3, Additional file 2: Dataset S1.7). From the expression pattern for ERFs in our study, we found that more than half of them were up-regulated under cold treatment, with 44 in K326 and 27 in CB-1 (Additional file 1: Table S3). The most highly responsive ERFs are members of ABR (c74525_g2: around 40 times up-regulation) and CBF (c64865_g1: around 30 times up-regulation). These results suggest that ERFs may be involved in plant response to cold stress as regulated by ethylene levels.

Different from ethylene, salicylic acid (SA) shows a different pattern in CB-1 vs. K326, with up-regulation in CB-1 and down-regulation in K326. Previous studies showed that cold temperatures promote the accumulation of endogenous free SA and glucosyl SA in *Arabidopsis* shoots, wheat, and grape berry [28, 59, 63, 64]. Additionally, it has been reported that high concentrations and the continual application of SA cause severe growth damage and a decrease in cold tolerance capacity [59, 65–67]. Plants from seeds imbibed in a high concentration of SA (1 mM) did not show any alternation of cold tolerance, whereas low concentrations of SA (0.1–0.5 mM) promoted tolerance to cold stress in bean and tomato [68]. In summary, these data suggest that the sporadic application of SA may enhance the cold tolerance but that continual application may decrease this tolerance. Hence, the increasing of SA in CB-1 may be another reason for its relatively weaker cold tolerance. SA is synthesized via two distinct pathways, the isochorismate (IC) pathway and the phenylalnine ammonia-lyase (PAL) pathway [67]. Although we do not yet have direct measurements of the level of chorismate, many secondary metabolites were up-regulated in CB-1, which may be an upstream indication relating to the high level of SA in CB-1. These results suggest that the effect of SA on cold tolerance may be dependent on the organism, concentration, and period of application.

## Conclusions

CB-1 and K326 are two closely related tobacco cultivars, but their tolerance to cold is quite different. In summary, the large-scale-“omics” technologies utilized here to investigate the overall transcriptome and metabolome changes associated with cold treatment are surprisingly informative in uncovering novel regulatory elements and genes. Consequently, we found that transcriptional regulators/TFs, which are persistently changed during cold treatment, can be responsible for maintaining the cold acclimated status via complex regulatory networks. Moreover, both the transcriptomes and metabolomes indicated the higher responsive ability on common genes/metabolites might be one of the major reasons to explain the cold tolerance for K326. In addition, there may exist other cold regulatory pathways in K326 that are inactive or not present in CB-1. The results of our study contribute to a deeper understanding of the highly complex regulatory mechanisms that function in plants during cold treatment.

## Additional files

Additional file 1: Figure S1. The expression levels for cold responsive genes *ICE1* under cold treatment. 1: 2 h control; 2: 2 h treatment; 3: 6 h control; 4: 6 h treatment; 5: 12 h control; 6: 12 h treatment; 7: 1d control; 8: 1d treatment; 9: 2d control; 10: 2d treatment; 11: 3d control; 12: 3d treatment; 13: 4d control; 14: 4d treatment. **Figure S2**. Gene classification based on gene ontology (GO) for differentially expressed genes by cold up-regulated (induced) gene and cold down-regulated (repressed) gene. Blue color represents enriched GO terms by cold induced genes; Red color represents enriched GO terms by cold repressed genes. **Figure S3**. Gene classification based on gene ontology (GO) for differentially expressed genes (DEGs) of CB-1 and K326. Blue color represents the enriched GO terms based on DEGs of CB-1. Red color represents the enriched GO terms based on DEGs of K326. **Figure S4**. Boxplot for levels of secondary metabolites in CC, CT, KC and KT. **Figure S5**. Visualization of the final gene-metabolite network. Blue color represents genes; Red color represents secondary metabolites; cyan color represents primary metabolites. **Figure S6**. Visualization of interaction networks of sugar metabolites and identified secondary metabolites. (A) The interaction networks of sugar metabolites and associated genes. (B) The interaction networks of identified secondary metabolites and associated genes. **Table S1**. Primer sequences used for qRT-PCR analysis. **Table S2**. The detailed expression levels for known cold responsive genes and DREB family genes. **Table S3**. The number of up-regulated, down-regulated and unchanged genes for ethylene biosynthesis family. (PPTX 788 kb)

Additional file 2: Dataset S1. RNA-seq assembly results and expression analysis from various tissues. (XLSX 38411 kb)

## Abbreviations

ABA: Abscisic acid
CBF: CCAAT-binding transcription factor
CRT: C-repeat
DEG: Differentially expressed gene
DREB: Dehydration responsive element
EIN: Ethylene insensitive protein
ERF: Ethylene responsive transcription factor
ET: Ethylene
GA: Gibberellin
GO: Gene ontology
IC: Isochorismate
ICE1: Inducer of CBF Expression 1
KEGG: Kyoto Encyclopedia of Genes and Genomes
PAL: Phenylalnine ammonia lyase
SA: Salicylic acid

## References

[1] Chinnusamy V, Zhu J, Zhu JK (2007). Cold stress regulation of gene expression in plants. Trends Plant Sci.

[2] Jeon J, Kim J (2013). Cold stress signaling networks in Arabidopsis. Journal of Plant Biology.

[3] Xiong L, Schumaker KS, Zhu JK (2002). Cell signaling during cold, drought, and salt stress. Plant Cell.

[4] Sanghera GS, Wani SH, Hussain W, Singh NB (2011). Engineering cold stress tolerance in crop plants. Curr Genomics.

[5] Yang QS, Gao J, He WD, Dou TX, Ding LJ, Wu JH, Li CY, Peng XX, Zhang S, Yi GJ (2015). Comparative transcriptomics analysis reveals difference of key gene expression between banana and plantain in response to cold stress. BMC Genomics.

[6] Zhang JZ, Creelman RA, Zhu JK (2004). From laboratory to field. Using information from Arabidopsis to engineer salt, cold, and drought tolerance in crops. Plant Physiol.

[7] Bielecka M, Watanabe M, Morcuende R, Scheible WR, Hawkesford MJ, Hesse H, Hoefgen R (2014). Transcriptome and metabolome analysis of plant sulfate starvation and resupply provides novel information on transcriptional regulation of metabolism associated with sulfur, nitrogen and phosphorus nutritional responses in Arabidopsis. Front Plant Sci.

[8] Tohge T, Nishiyama Y, Hirai MY, Yano M, Nakajima J, Awazuhara M, Inoue E, Takahashi H, Goodenowe DB, Kitayama M (2005). Functional genomics by integrated analysis of metabolome and transcriptome of Arabidopsis plants over-expressing an MYB transcription factor. Plant J.

[9] Jin J, Kim MJ, Dhandapani S, Tjhang JG, Yin JL, Wong L, Sarojam R, Chua NH, Jang IC (2015). The floral transcriptome of ylang ylang (*Cananga odorata* Var. Fruticosa) uncovers biosynthetic pathways for volatile organic compounds and a multifunctional and novel sesquiterpene synthase. J Exp Bot.

[10] Jin J, Panicker D, Wang Q, Kim MJ, Liu J, Yin JL, Wong L, Jang IC, Chua NH, Sarojam R (2014). Next generation sequencing unravels the biosynthetic ability of spearmint (*Mentha spicata*) peltate glandular trichomes through comparative transcriptomics. BMC Plant Biol.

[11] Kim MJ, Jin J, Zheng J, Wong L, Chua NH, Jang IC (2015). Comparative Transcriptomics unravel biochemical specialization of leaf tissues of Stevia for Diterpenoid production. Plant Physiol.

[12] Calzadilla PI, Maiale SJ, Ruiz OA, Escaray FJ (2016). Transcriptome response mediated by cold stress in Lotus Japonicus. Front Plant Sci.

[13] Tian DQ, Pan XY, Yu YM, Wang WY, Zhang F, Ge YY, Shen XL, Shen FQ, Liu XJ (2013). De novo characterization of the Anthurium transcriptome and analysis of its digital gene expression under cold stress. BMC Genomics.

[14] Hwang I, Manoharan RK, Kang JG, Chung MY, Kim YW, Nou IS (2016). Genome-wide identification and characterization of bZIP transcription factors in *Brassica oleracea* under cold stress. Biomed Res Int.

[15] Lei X, Xiao Y, Xia W, Mason AS, Yang Y, Ma Z, Peng M (2014). RNA-seq analysis of oil palm under cold stress reveals a different C-repeat binding factor (CBF) mediated gene expression pattern in *Elaeis guineensis* compared to other species. PLoS One.

[16] Zhang Y, Wu H, Xie J, Jiang R, Deng C, Pang H (2015). Transcriptome responses to heat- and cold-stress in ladybirds (*Cryptolaemus montrouzieri* Mulasnt) analyzed by deep-sequencing. Biol Res.

[17] Bai B, Wu J, Sheng WT, Zhou B, Zhou LJ, Zhuang W, Yao DP, Deng QY (2015). Comparative analysis of anther Transcriptome profiles of two different Rice male sterile lines genotypes under cold stress. Int J Mol Sci.

[18] Zhao L, Liu F, Xu W, Di C, Zhou S, Xue Y, Yu J, Su Z (2009). Increased expression of OsSPX1 enhances cold/subfreezing tolerance in tobacco and *Arabidopsis thaliana*. Plant Biotechnol J.

[19] Maruyama K, Urano K, Yoshiwara K, Morishita Y, Sakurai N, Suzuki H, Kojima M, Sakakibara H, Shibata D, Saito K (2014). Integrated analysis of the effects of cold and dehydration on rice metabolites, phytohormones, and gene transcripts. Plant Physiol.

[20] Chen JQ, Dong Y, Wang YJ, Liu Q, Zhang JS, Chen SY (2003). An AP2/EREBP-type transcription-factor gene from rice is cold-inducible and encodes a nuclear-localized protein. Theor Appl Genet.

[21] He Y, Li Y, Cui L, Xie L, Zheng C, Zhou G, Zhou J, Xie X (2016). Phytochrome B negatively affects cold tolerance by regulating OsDREB1 Gene expression through Phytochrome interacting factor-like protein OsPIL16 in Rice. Front Plant Sci.

[22] Xiaochuang C, Chu Z, Lianfeng Z, Junhua Z, Hussain S, Lianghuan W, Qianyu J (2017). Glycine increases cold tolerance in rice via the regulation of N uptake, physiological characteristics, and photosynthesis. Plant Physiol Biochem.

[23] Bilska-Kos A, Szczepanik J, Sowinski P (2016). Cold induced changes in the water balance affect immunocytolocalization pattern of one of the aquaporins in the vascular system in the leaves of maize (*Zea mays* L.). J Plant Physiol.

[24] Riva-Roveda L, Escale B, Giauffret C, Perilleux C (2016). Maize plants can enter a standby mode to cope with chilling stress. BMC Plant Biol.

[25] Bustamante CA, Monti LL, Gabilondo J, Scossa F, Valentini G, Budde CO, Lara MV, Fernie AR, Drincovich MF (2016). Differential metabolic rearrangements after cold storage are correlated with chilling injury resistance of peach fruits. Front Plant Sci.

[26] Sanhueza D, Vizoso P, Balic I, Campos-Vargas R, Meneses C (2015). Transcriptomic analysis of fruit stored under cold conditions using controlled atmosphere in *Prunus persica* cv. “red pearl”. Front Plant Sci.

[27] Zhao M, Liu W, Xia X, Wang T, Zhang WH (2014). Cold acclimation-induced freezing tolerance of *Medicago truncatula* seedlings is negatively regulated by ethylene. Physiol Plant.

[28] Kosova K, Prasil IT, Vitamvas P, Dobrev P, Motyka V, Flokova K, Novak O, Tureckova V, Rolcik J, Pesek B (2012). Complex phytohormone responses during the cold acclimation of two wheat cultivars differing in cold tolerance, winter Samanta and spring Sandra. J Plant Physiol.

[29] Khodakovskaya M, McAvoy R, Peters J, Wu H, Li Y (2006). Enhanced cold tolerance in transgenic tobacco expressing a chloroplast omega-3 fatty acid desaturase gene under the control of a cold-inducible promoter. Planta.

[30] Liu X, Shi W, Yin W, Wang J (2016). Distinct cold responsiveness of a StInvInh2 gene promoter in transgenic potato tubers with contrasting resistance to cold-induced sweetening. Plant Physiol Biochem.

[31] Cook D, Fowler S, Fiehn O, Thomashow MF (2004). A prominent role for the CBF cold response pathway in configuring the low-temperature metabolome of Arabidopsis. Proc Natl Acad Sci U S A.

[32] Riechmann JL, Meyerowitz EM (1998). The AP2/EREBP family of plant transcription factors. Biol Chem.

[33] Wu ZG, Jiang W, Chen SL, Mantri N, Tao ZM, Jiang CX (2016). Insights from the cold Transcriptome and Metabolome of Dendrobium Officinale: global reprogramming of metabolic and Gene regulation networks during cold acclimation. Front Plant Sci.

[34] Kaplan F, Kopka J, Haskell DW, Zhao W, Schiller KC, Gatzke N, Sung DY, Guy CL (2004). Exploring the temperature-stress metabolome of Arabidopsis. Plant Physiol.

[35] Sun X, Zhao T, Gan S, Ren X, Fang L, Karungo SK, Wang Y, Chen L, Li S, Xin H (2016). Ethylene positively regulates cold tolerance in grapevine by modulating the expression of ETHYLENE RESPONSE FACTOR 057. Sci Rep.

[36] Gao W, Sun HX, Xiao H, Cui G, Hillwig ML, Jackson A, Wang X, Shen Y, Zhao N, Zhang L (2014). Combining metabolomics and transcriptomics to characterize tanshinone biosynthesis in salvia miltiorrhiza. BMC Genomics.

[37] Kaplan F, Kopka J, Sung DY, Zhao W, Popp M, Porat R, Guy CL (2007). Transcript and metabolite profiling during cold acclimation of Arabidopsis reveals an intricate relationship of cold-regulated gene expression with modifications in metabolite content. Plant J.

[38] Hirai MY, Sugiyama K, Sawada Y, Tohge T, Obayashi T, Suzuki A, Araki R, Sakurai N, Suzuki H, Aoki K (2007). Omics-based identification of Arabidopsis Myb transcription factors regulating aliphatic glucosinolate biosynthesis. Proc Natl Acad Sci U S A.

[39] Jin CZY (2011). Proteomic analysis of cold stress responses in tobacco seedlings. Afr J Biotechnol.

[40] Zhang Z, Huang R (2010). Enhanced tolerance to freezing in tobacco and tomato overexpressing transcription factor TERF2/LeERF2 is modulated by ethylene biosynthesis. Plant Mol Biol.

[41] Fumagalli M, Vieira FG, Linderoth T, Nielsen R (2014). ngsTools: methods for population genetics analyses from next-generation sequencing data. Bioinformatics.

[42] Grabherr MG, Haas BJ, Yassour M, Levin JZ, Thompson DA, Amit I, Adiconis X, Fan L, Raychowdhury R, Zeng Q (2011). Full-length transcriptome assembly from RNA-Seq data without a reference genome. Nat Biotechnol.

[43] UniProt C (2015). UniProt: a hub for protein information. Nucleic Acids Res.

[44] Islamaj Dogan R, Kim S, Chatr-Aryamontri A, Chang CS, Oughtred R, Rust J, Wilbur WJ, Comeau DC, Dolinski K, Tyers M (2017). The BioC-BioGRID corpus: full text articles annotated for curation of protein-protein and genetic interactions. Database (Oxford).

[45] Hermjakob H, Montecchi-Palazzi L, Lewington C, Mudali S, Kerrien S, Orchard S, Vingron M, Roechert B, Roepstorff P, Valencia A (2004). IntAct: an open source molecular interaction database. Nucleic Acids Res.

[46] Boue S, Talikka M, Westra JW, Hayes W, Di Fabio A, Park J, Schlage WK, Sewer A, Fields B, Ansari S (2015). Causal biological network database: a comprehensive platform of causal biological network models focused on the pulmonary and vascular systems. Database (Oxford).

[47] Xenarios I, Salwinski L, Duan XJ, Higney P, Kim SM, Eisenberg D (2002). DIP, the database of interacting proteins: a research tool for studying cellular networks of protein interactions. Nucleic Acids Res.

[48] Chatr-aryamontri A, Ceol A, Palazzi LM, Nardelli G, Schneider MV, Castagnoli L, Cesareni G (2007). MINT: the molecular INTeraction database. Nucleic Acids Res.

[49] Goel R, Harsha HC, Pandey A, Prasad TS (2012). Human protein reference database and human Proteinpedia as resources for phosphoproteome analysis. Mol BioSyst.

[50] Mewes HW, Frishman D, Mayer KF, Munsterkotter M, Noubibou O, Pagel P, Rattei T, Oesterheld M, Ruepp A, Stumpflen V (2006). MIPS: analysis and annotation of proteins from whole genomes in 2005. Nucleic Acids Res.

[51] Lamesch P, Berardini TZ, Li D, Swarbreck D, Wilks C, Sasidharan R, Muller R, Dreher K, Alexander DL, Garcia-Hernandez M (2012). The Arabidopsis information resource (TAIR): improved gene annotation and new tools. Nucleic Acids Res.

[52] Shannon P, Markiel A, Ozier O, Baliga NS, Wang JT, Ramage D, Amin N, Schwikowski B, Ideker T (2003). Cytoscape: a software environment for integrated models of biomolecular interaction networks. Genome Res.

[53] Mount DW (2007). Using the basic local alignment search tool (BLAST). CSH Protoc.

[54] Langmead B, Salzberg SL (2012). Fast gapped-read alignment with bowtie 2. Nat Methods.

[55] Li B, Dewey CN (2011). RSEM: accurate transcript quantification from RNA-Seq data with or without a reference genome. BMC Bioinformatics.

[56] Mitchell A, Chang HY, Daugherty L, Fraser M, Hunter S, Lopez R, McAnulla C, McMenamin C, Nuka G, Pesseat S (2015). The InterPro protein families database: the classification resource after 15 years. Nucleic Acids Res.

[57] Chen H, Chen X, Chen D, Li J, Zhang Y, Wang A (2015). A comparison of the low temperature transcriptomes of two tomato genotypes that differ in freezing tolerance: *Solanum lycopersicum* and *Solanum habrochaites*. BMC Plant Biol.

[58] Mousavi S, Alisoltani A, Shiran B, Fallahi H, Ebrahimie E, Imani A, Houshmand S (2014). De novo transcriptome assembly and comparative analysis of differentially expressed genes in *Prunus dulcis* mill. In response to freezing stress. PLoS One.

[59] Miura K, Tada Y (2014). Regulation of water, salinity, and cold stress responses by salicylic acid. Front Plant Sci.

[60] Catala R, Lopez-Cobollo R, Mar Castellano M, Angosto T, Alonso JM, Ecker JR, Salinas J (2014). The Arabidopsis 14-3-3 protein RARE COLD INDUCIBLE 1A links low-temperature response and ethylene biosynthesis to regulate freezing tolerance and cold acclimation. Plant Cell.

[61] Shi Y, Tian S, Hou L, Huang X, Zhang X, Guo H, Yang S (2012). Ethylene signaling negatively regulates freezing tolerance by repressing expression of CBF and type-a ARR genes in Arabidopsis. Plant Cell.

[62] Ciardi JA, Deikman J, Orzolek MD (1997). Increased ethylene synthesis enhances chilling tolerance in tomato. Physiol Plant.

[63] Scott IM, Clarke SM, Wood JE, Mur LA (2004). Salicylate accumulation inhibits growth at chilling temperature in Arabidopsis. Plant Physiol.

[64] Wan SB, Tian L, Tian RR, Pan QH, Zhan JC, Wen PF, Chen JY, Zhang P, Wang W, Huang WD (2009). Involvement of phospholipase D in the low temperature acclimation-induced thermotolerance in grape berry. Plant Physiol Biochem.

[65] Bowling SA, Clarke JD, Liu Y, Klessig DF, Dong X (1997). The cpr5 mutant of Arabidopsis expresses both NPR1-dependent and NPR1-independent resistance. Plant Cell.

[66] Rate DN, Cuenca JV, Bowman GR, Guttman DS, Greenberg JT (1999). The gain-of-function Arabidopsis acd6 mutant reveals novel regulation and function of the salicylic acid signaling pathway in controlling cell death, defenses, and cell growth. Plant Cell.

[67] Lee J, Nam J, Park HC, Na G, Miura K, Jin JB, Yoo CY, Baek D, Kim DH, Jeong JC (2007). Salicylic acid-mediated innate immunity in Arabidopsis is regulated by SIZ1 SUMO E3 ligase. Plant J.

[68] Yuan S, Lin HH (2008). Role of salicylic acid in plant abiotic stress. Z Naturforsch C.

