# Supplementary material for: Integrated transcriptomics and metabolomics analysis to characterize cold stress responses in Nicotiana tabacum
Source: BMC Genomics. 2017 Jun 29;18:496. doi: 10.1186/s12864-017-3871-7 (PMC5492280; doi:10.1186/s12864-017-3871-7)
Supplement: Supplementary file 1 — The expression levels for cold responsive genes ICE1 under cold treatment. 1: 2 h control; 2: 2 h treatment; 3: 6 h control; 4: 6 h treatment; 5: 12 h control; 6: 12 h treatment; 7: 1d control; 8: 1d treatment; 9: 2d control; 10: 2d treatment; 11: 3d control; 12: 3d treatment; 13: 4d control; 14: 4d treatment. Figure S2. Gene classification based on gene ontology (GO) for differentially expressed genes by cold up-regulated (induced) gene and cold down-regulated (repressed) gene. Blue color represents enriched GO terms by cold induced genes; Red color represents enriched GO terms by cold repressed genes. Figure S3. Gene classification based on gene ontology (GO) for differentially expressed genes (DEGs) of CB-1 and K326. Blue color represents the enriched GO terms based on DEGs of CB-1. Red color represents the enriched GO terms based on DEGs of K326. Figure S4. Boxplot for levels of secondary metabolites in CC, CT, KC and KT. Figure S5. Visualization of the final gene-metabolite network. Blue color represents genes; Red color represents secondary metabolites; cyan color represents primary metabolites. Figure S6. Visualization of interaction networks of sugar metabolites and identified secondary metabolites. (A) The interaction networks of sugar metabolites and associated genes. (B) The interaction networks of identified secondary metabolites and associated genes. Table S1. Primer sequences used for qRT-PCR analysis. Table S2. The detailed expression levels for known cold responsive genes and DREB family genes. Table S3. The number of up-regulated, down-regulated and unchanged genes for ethylene biosynthesis family. (PPTX 788 kb) [file 12864_2017_3871_MOESM1_ESM.pptx]

## Slide 1
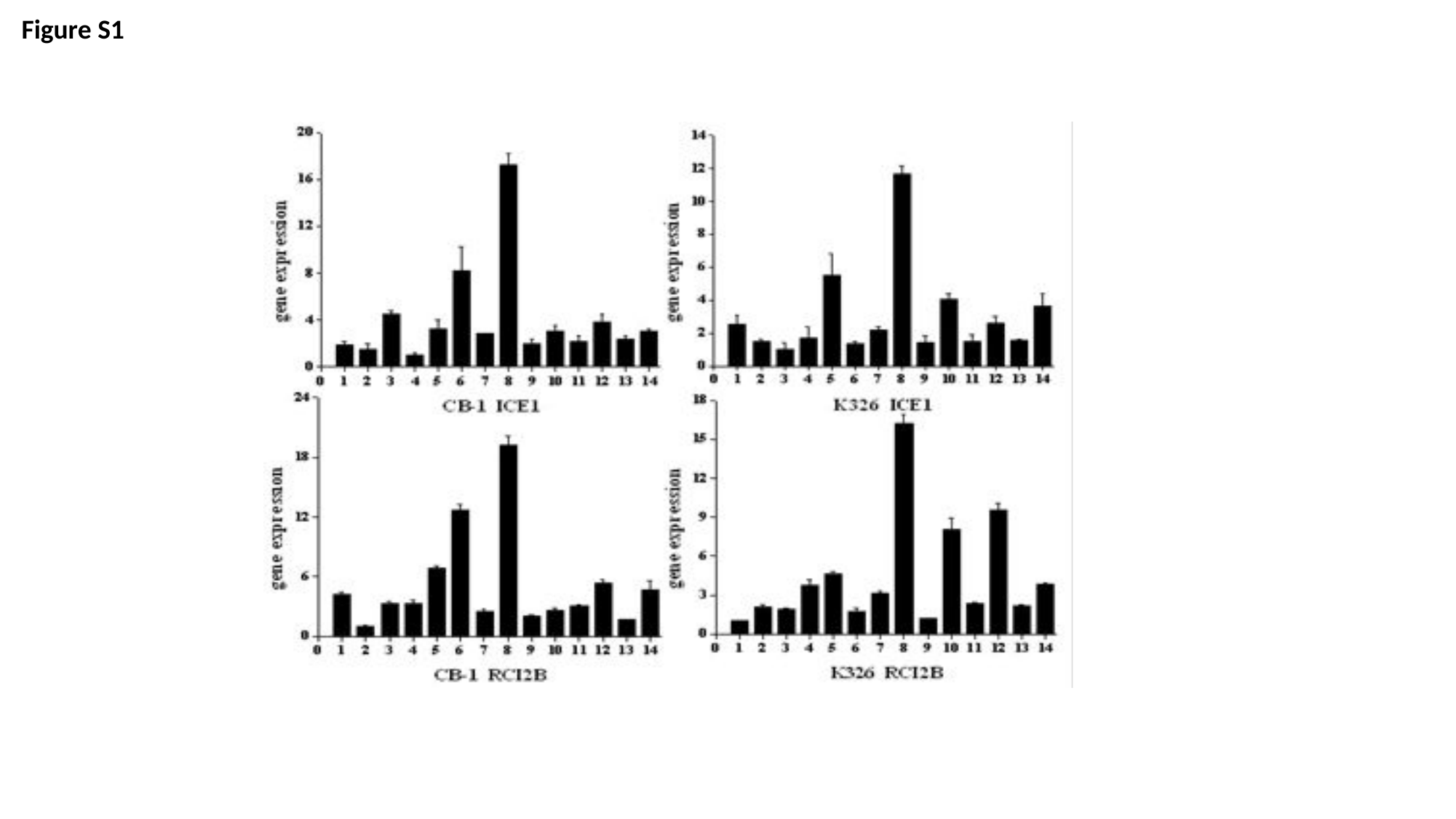

Figure S1

## Slide 2
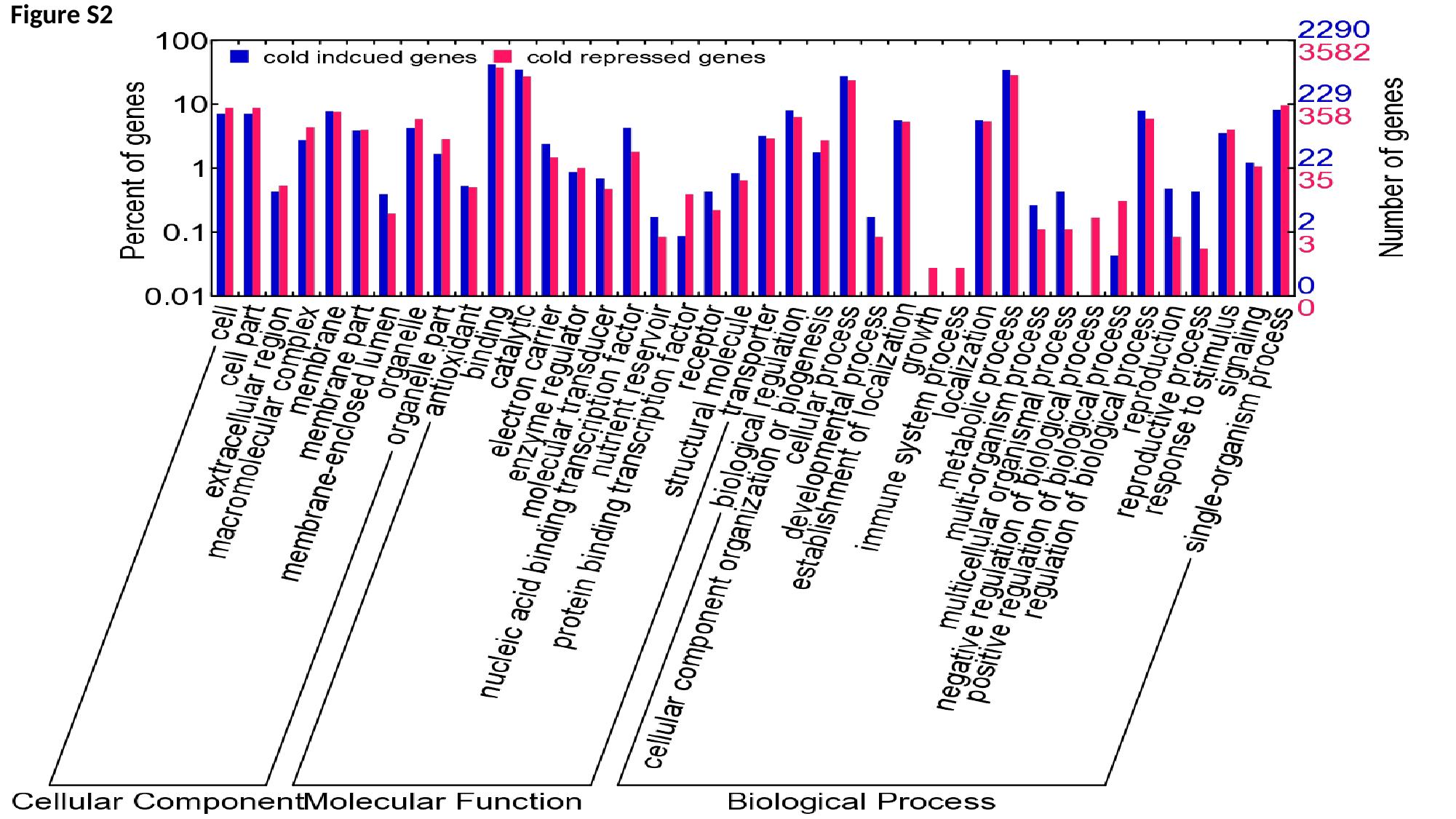

Figure S2

## Slide 3
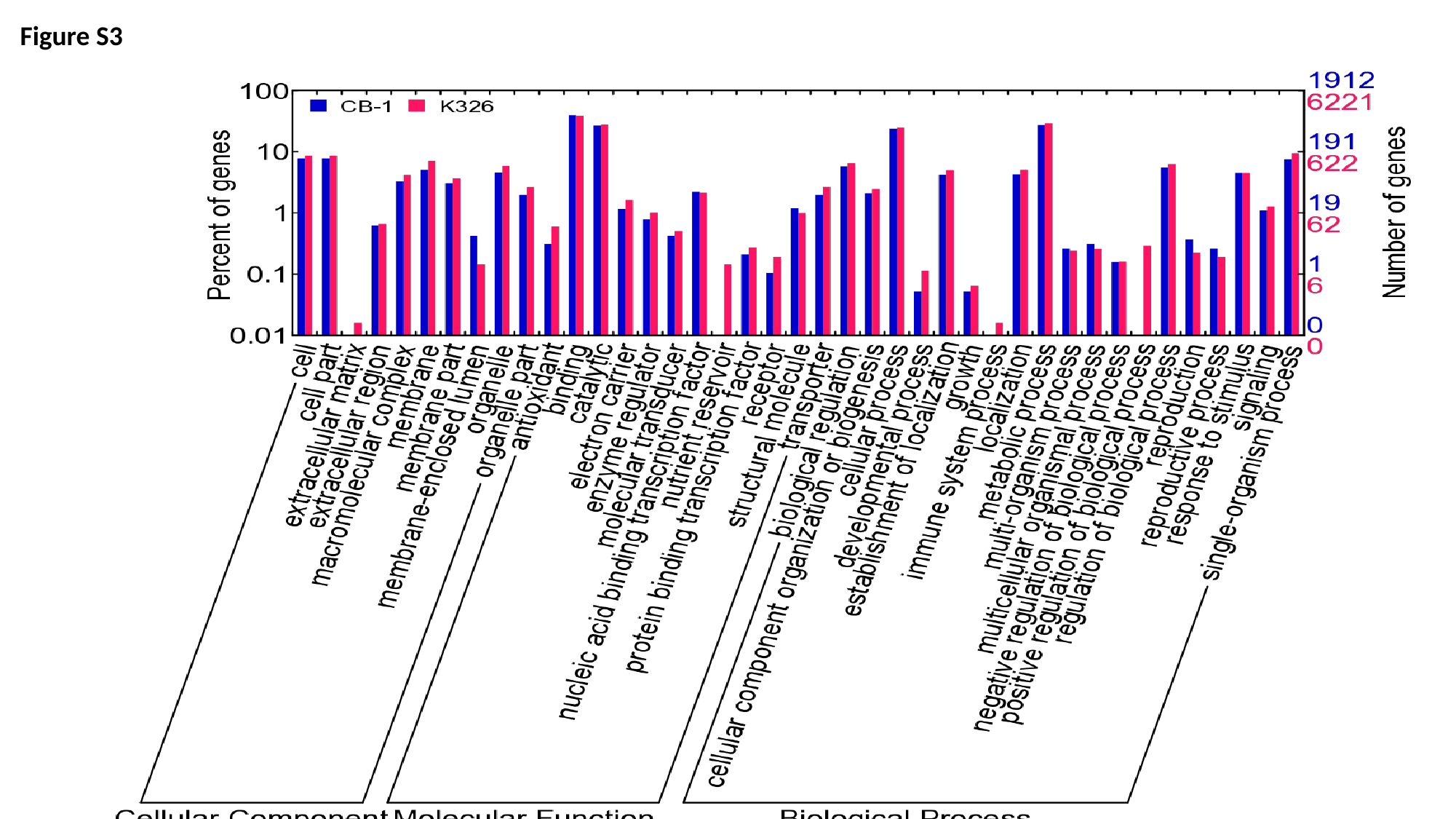

Figure S3

## Slide 4
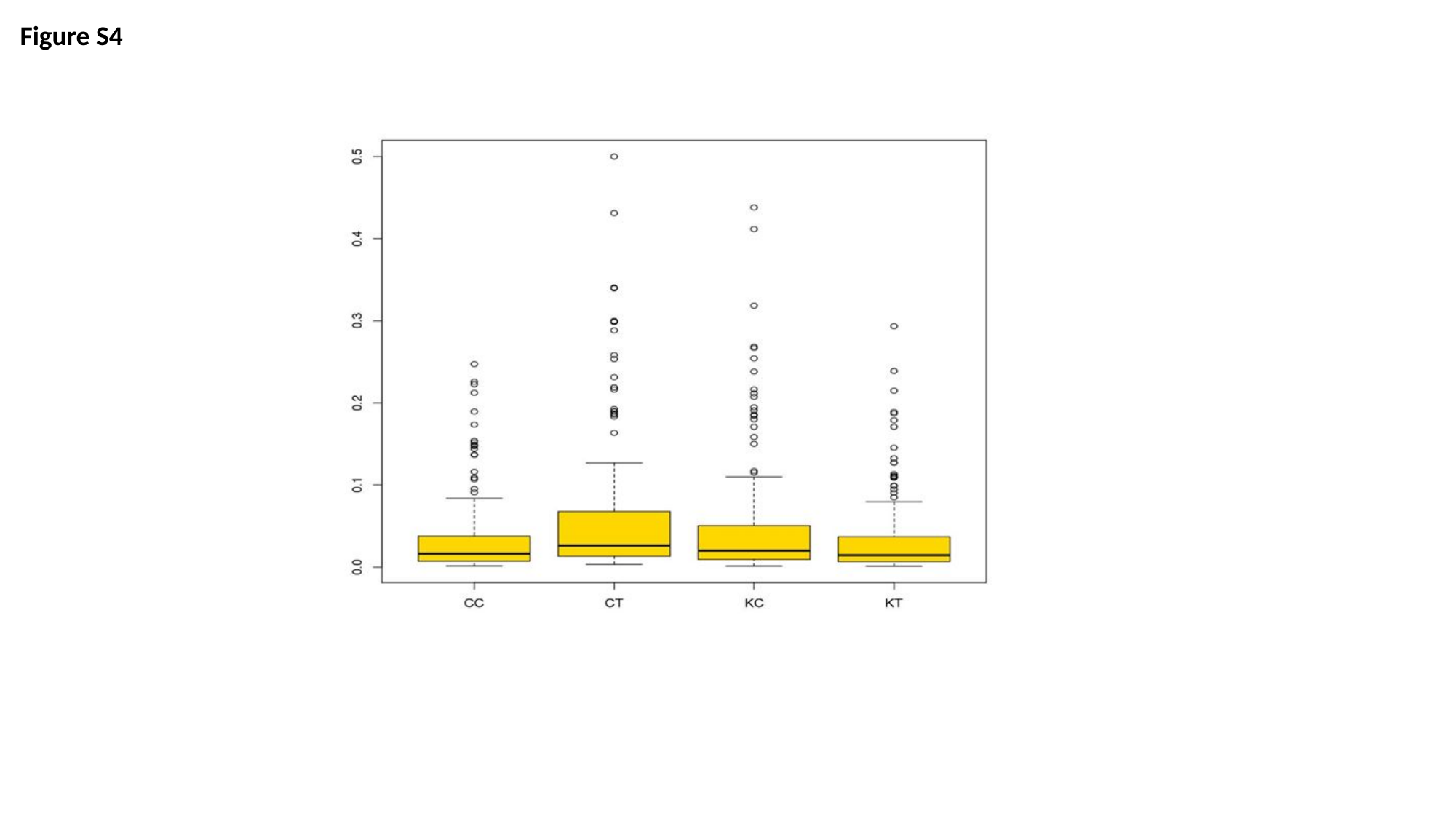

Figure S4

## Slide 5
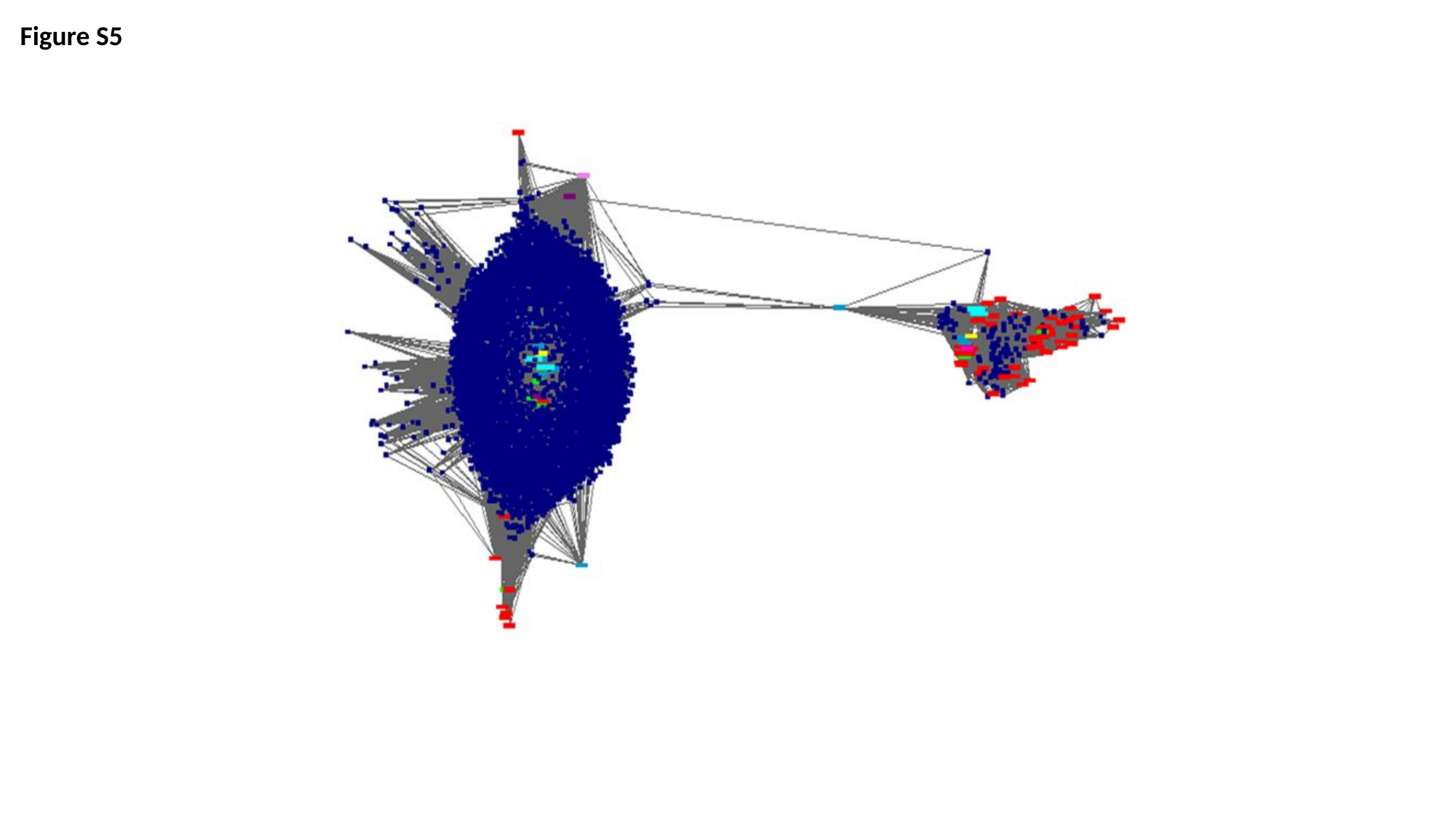

Figure S5

## Slide 6
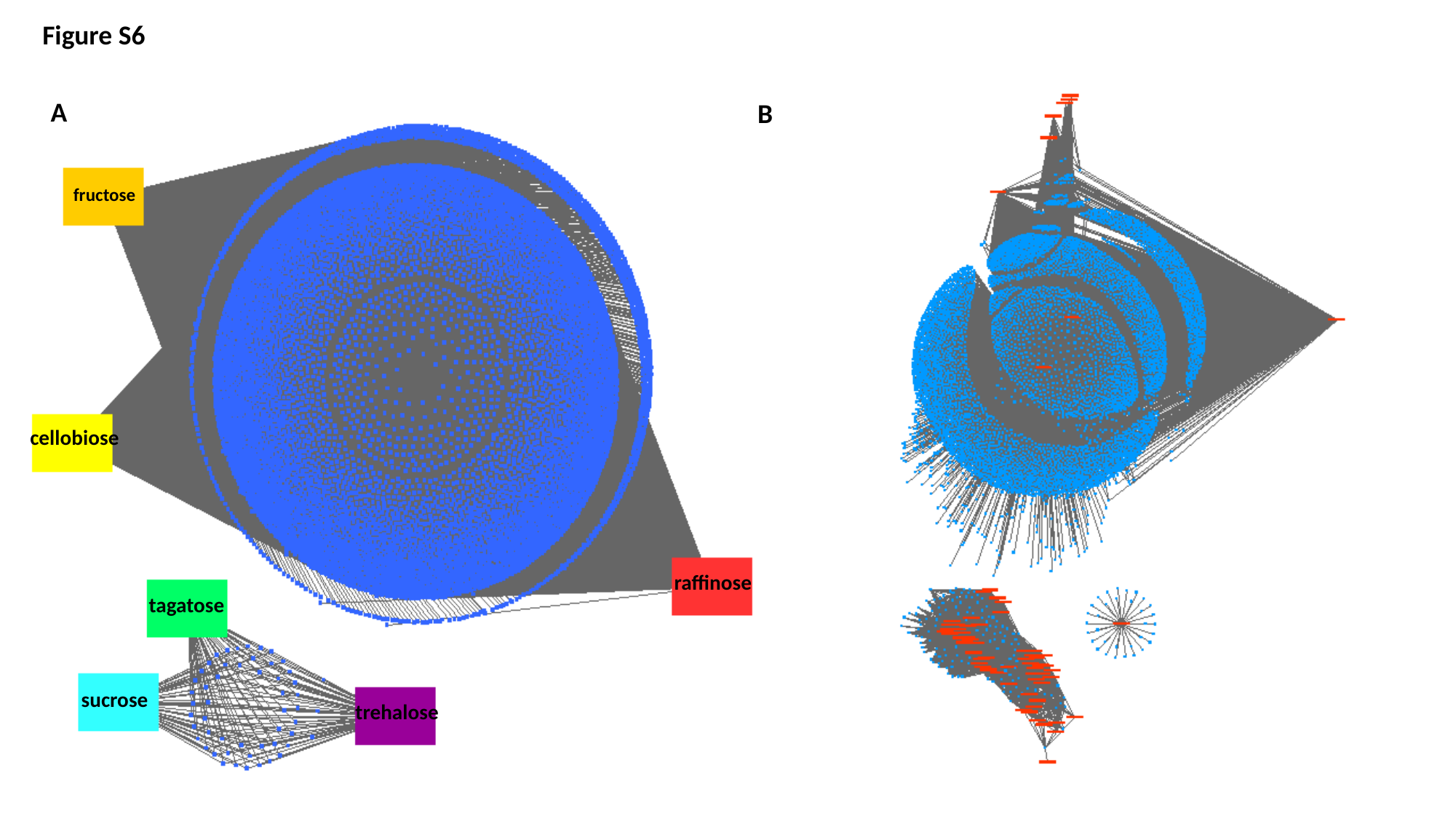

Figure S6
A
B
fructose
cellobiose
raffinose
tagatose
sucrose
trehalose

## Slide 7
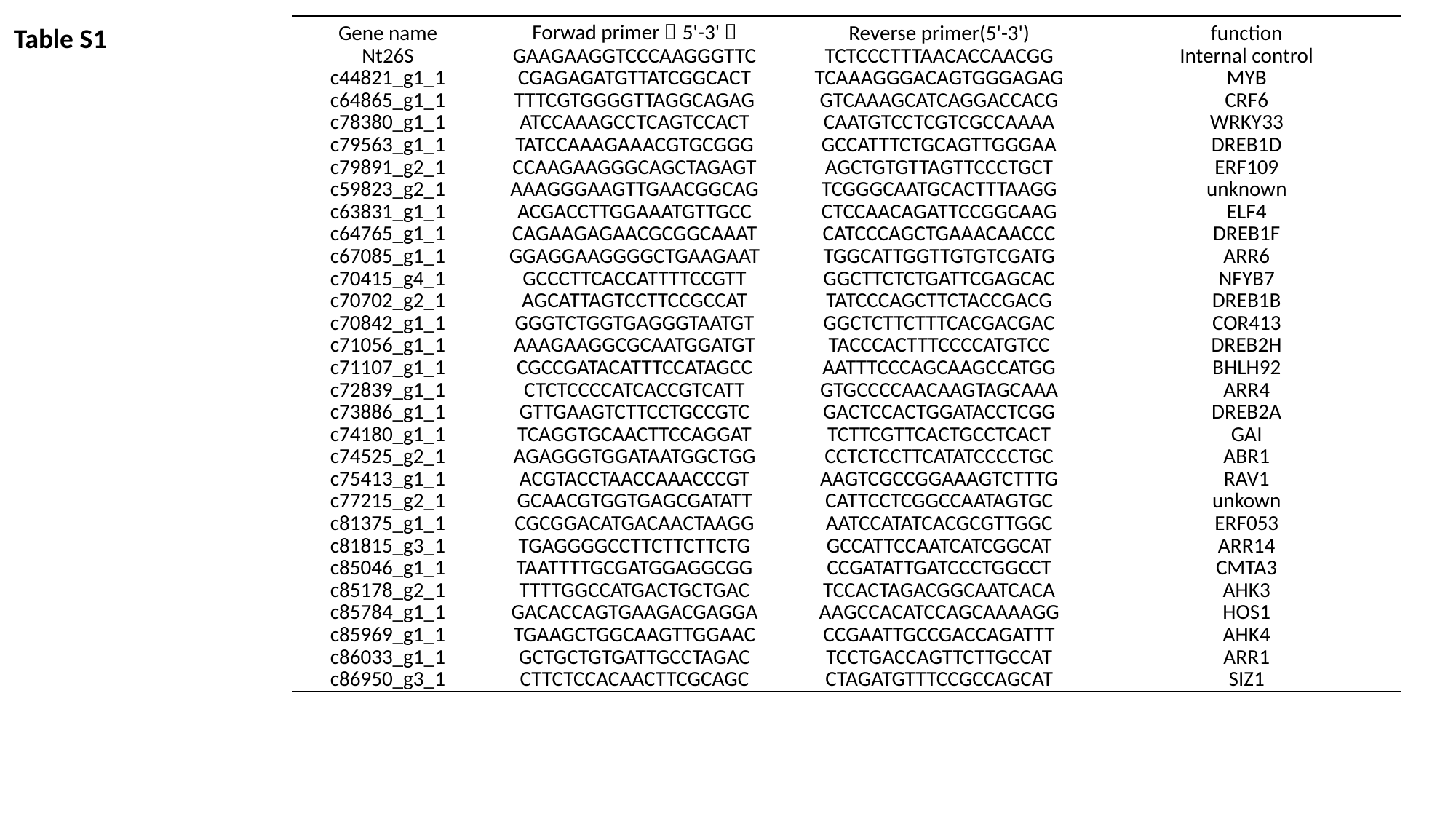

Table S1
| Gene name | Forwad primer（5'-3'） | Reverse primer(5'-3') | function |
| --- | --- | --- | --- |
| Nt26S | GAAGAAGGTCCCAAGGGTTC | TCTCCCTTTAACACCAACGG | Internal control |
| c44821\_g1\_1 | CGAGAGATGTTATCGGCACT | TCAAAGGGACAGTGGGAGAG | MYB |
| c64865\_g1\_1 | TTTCGTGGGGTTAGGCAGAG | GTCAAAGCATCAGGACCACG | CRF6 |
| c78380\_g1\_1 | ATCCAAAGCCTCAGTCCACT | CAATGTCCTCGTCGCCAAAA | WRKY33 |
| c79563\_g1\_1 | TATCCAAAGAAACGTGCGGG | GCCATTTCTGCAGTTGGGAA | DREB1D |
| c79891\_g2\_1 | CCAAGAAGGGCAGCTAGAGT | AGCTGTGTTAGTTCCCTGCT | ERF109 |
| c59823\_g2\_1 | AAAGGGAAGTTGAACGGCAG | TCGGGCAATGCACTTTAAGG | unknown |
| c63831\_g1\_1 | ACGACCTTGGAAATGTTGCC | CTCCAACAGATTCCGGCAAG | ELF4 |
| c64765\_g1\_1 | CAGAAGAGAACGCGGCAAAT | CATCCCAGCTGAAACAACCC | DREB1F |
| c67085\_g1\_1 | GGAGGAAGGGGCTGAAGAAT | TGGCATTGGTTGTGTCGATG | ARR6 |
| c70415\_g4\_1 | GCCCTTCACCATTTTCCGTT | GGCTTCTCTGATTCGAGCAC | NFYB7 |
| c70702\_g2\_1 | AGCATTAGTCCTTCCGCCAT | TATCCCAGCTTCTACCGACG | DREB1B |
| c70842\_g1\_1 | GGGTCTGGTGAGGGTAATGT | GGCTCTTCTTTCACGACGAC | COR413 |
| c71056\_g1\_1 | AAAGAAGGCGCAATGGATGT | TACCCACTTTCCCCATGTCC | DREB2H |
| c71107\_g1\_1 | CGCCGATACATTTCCATAGCC | AATTTCCCAGCAAGCCATGG | BHLH92 |
| c72839\_g1\_1 | CTCTCCCCATCACCGTCATT | GTGCCCCAACAAGTAGCAAA | ARR4 |
| c73886\_g1\_1 | GTTGAAGTCTTCCTGCCGTC | GACTCCACTGGATACCTCGG | DREB2A |
| c74180\_g1\_1 | TCAGGTGCAACTTCCAGGAT | TCTTCGTTCACTGCCTCACT | GAI |
| c74525\_g2\_1 | AGAGGGTGGATAATGGCTGG | CCTCTCCTTCATATCCCCTGC | ABR1 |
| c75413\_g1\_1 | ACGTACCTAACCAAACCCGT | AAGTCGCCGGAAAGTCTTTG | RAV1 |
| c77215\_g2\_1 | GCAACGTGGTGAGCGATATT | CATTCCTCGGCCAATAGTGC | unkown |
| c81375\_g1\_1 | CGCGGACATGACAACTAAGG | AATCCATATCACGCGTTGGC | ERF053 |
| c81815\_g3\_1 | TGAGGGGCCTTCTTCTTCTG | GCCATTCCAATCATCGGCAT | ARR14 |
| c85046\_g1\_1 | TAATTTTGCGATGGAGGCGG | CCGATATTGATCCCTGGCCT | CMTA3 |
| c85178\_g2\_1 | TTTTGGCCATGACTGCTGAC | TCCACTAGACGGCAATCACA | AHK3 |
| c85784\_g1\_1 | GACACCAGTGAAGACGAGGA | AAGCCACATCCAGCAAAAGG | HOS1 |
| c85969\_g1\_1 | TGAAGCTGGCAAGTTGGAAC | CCGAATTGCCGACCAGATTT | AHK4 |
| c86033\_g1\_1 | GCTGCTGTGATTGCCTAGAC | TCCTGACCAGTTCTTGCCAT | ARR1 |
| c86950\_g3\_1 | CTTCTCCACAACTTCGCAGC | CTAGATGTTTCCGCCAGCAT | SIZ1 |

## Slide 8
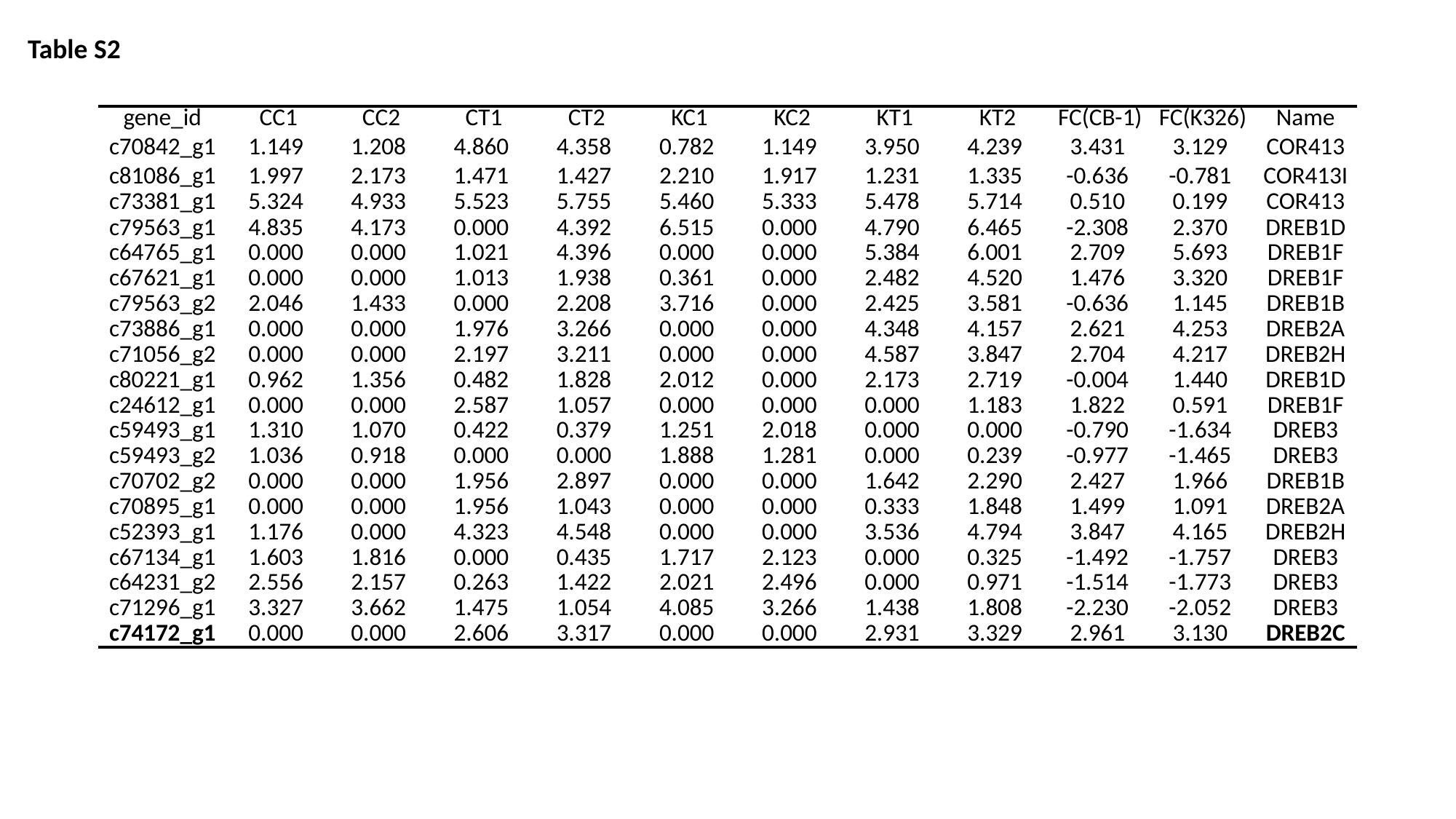

Table S2
| gene\_id | CC1 | CC2 | CT1 | CT2 | KC1 | KC2 | KT1 | KT2 | FC(CB-1) | FC(K326) | Name |
| --- | --- | --- | --- | --- | --- | --- | --- | --- | --- | --- | --- |
| c70842\_g1 | 1.149 | 1.208 | 4.860 | 4.358 | 0.782 | 1.149 | 3.950 | 4.239 | 3.431 | 3.129 | COR413 |
| c81086\_g1 | 1.997 | 2.173 | 1.471 | 1.427 | 2.210 | 1.917 | 1.231 | 1.335 | -0.636 | -0.781 | COR413I |
| c73381\_g1 | 5.324 | 4.933 | 5.523 | 5.755 | 5.460 | 5.333 | 5.478 | 5.714 | 0.510 | 0.199 | COR413 |
| c79563\_g1 | 4.835 | 4.173 | 0.000 | 4.392 | 6.515 | 0.000 | 4.790 | 6.465 | -2.308 | 2.370 | DREB1D |
| c64765\_g1 | 0.000 | 0.000 | 1.021 | 4.396 | 0.000 | 0.000 | 5.384 | 6.001 | 2.709 | 5.693 | DREB1F |
| c67621\_g1 | 0.000 | 0.000 | 1.013 | 1.938 | 0.361 | 0.000 | 2.482 | 4.520 | 1.476 | 3.320 | DREB1F |
| c79563\_g2 | 2.046 | 1.433 | 0.000 | 2.208 | 3.716 | 0.000 | 2.425 | 3.581 | -0.636 | 1.145 | DREB1B |
| c73886\_g1 | 0.000 | 0.000 | 1.976 | 3.266 | 0.000 | 0.000 | 4.348 | 4.157 | 2.621 | 4.253 | DREB2A |
| c71056\_g2 | 0.000 | 0.000 | 2.197 | 3.211 | 0.000 | 0.000 | 4.587 | 3.847 | 2.704 | 4.217 | DREB2H |
| c80221\_g1 | 0.962 | 1.356 | 0.482 | 1.828 | 2.012 | 0.000 | 2.173 | 2.719 | -0.004 | 1.440 | DREB1D |
| c24612\_g1 | 0.000 | 0.000 | 2.587 | 1.057 | 0.000 | 0.000 | 0.000 | 1.183 | 1.822 | 0.591 | DREB1F |
| c59493\_g1 | 1.310 | 1.070 | 0.422 | 0.379 | 1.251 | 2.018 | 0.000 | 0.000 | -0.790 | -1.634 | DREB3 |
| c59493\_g2 | 1.036 | 0.918 | 0.000 | 0.000 | 1.888 | 1.281 | 0.000 | 0.239 | -0.977 | -1.465 | DREB3 |
| c70702\_g2 | 0.000 | 0.000 | 1.956 | 2.897 | 0.000 | 0.000 | 1.642 | 2.290 | 2.427 | 1.966 | DREB1B |
| c70895\_g1 | 0.000 | 0.000 | 1.956 | 1.043 | 0.000 | 0.000 | 0.333 | 1.848 | 1.499 | 1.091 | DREB2A |
| c52393\_g1 | 1.176 | 0.000 | 4.323 | 4.548 | 0.000 | 0.000 | 3.536 | 4.794 | 3.847 | 4.165 | DREB2H |
| c67134\_g1 | 1.603 | 1.816 | 0.000 | 0.435 | 1.717 | 2.123 | 0.000 | 0.325 | -1.492 | -1.757 | DREB3 |
| c64231\_g2 | 2.556 | 2.157 | 0.263 | 1.422 | 2.021 | 2.496 | 0.000 | 0.971 | -1.514 | -1.773 | DREB3 |
| c71296\_g1 | 3.327 | 3.662 | 1.475 | 1.054 | 4.085 | 3.266 | 1.438 | 1.808 | -2.230 | -2.052 | DREB3 |
| c74172\_g1 | 0.000 | 0.000 | 2.606 | 3.317 | 0.000 | 0.000 | 2.931 | 3.329 | 2.961 | 3.130 | DREB2C |

## Slide 9
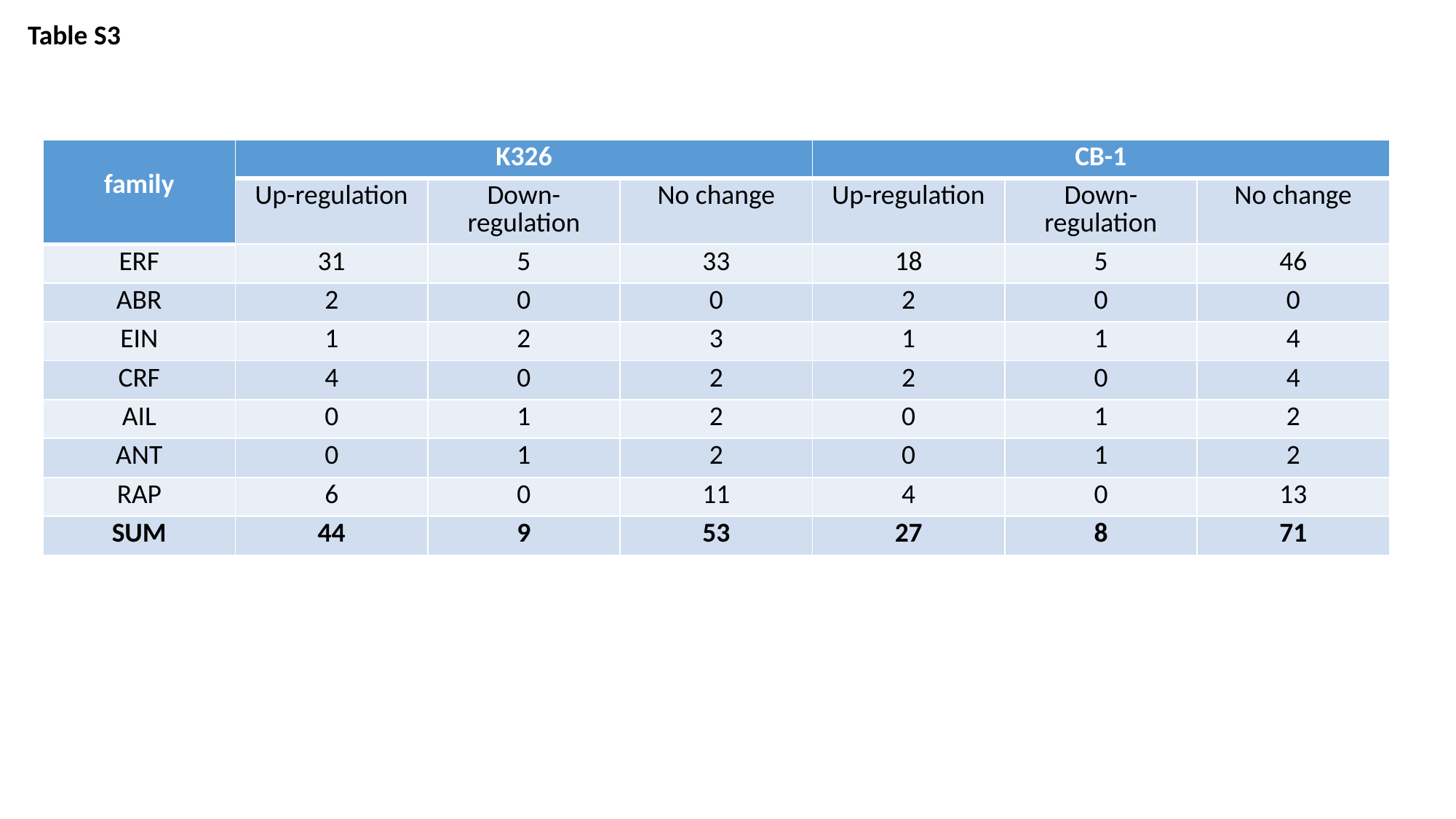

Table S3
| family | K326 | | | CB-1 | | |
| --- | --- | --- | --- | --- | --- | --- |
| | Up-regulation | Down-regulation | No change | Up-regulation | Down-regulation | No change |
| ERF | 31 | 5 | 33 | 18 | 5 | 46 |
| ABR | 2 | 0 | 0 | 2 | 0 | 0 |
| EIN | 1 | 2 | 3 | 1 | 1 | 4 |
| CRF | 4 | 0 | 2 | 2 | 0 | 4 |
| AIL | 0 | 1 | 2 | 0 | 1 | 2 |
| ANT | 0 | 1 | 2 | 0 | 1 | 2 |
| RAP | 6 | 0 | 11 | 4 | 0 | 13 |
| SUM | 44 | 9 | 53 | 27 | 8 | 71 |
